# SARD1 and CBP60g redundantly regulate N‐hydroxypipecolic acid‐ and salicylic acid‐dependent systemic immunity but are dispensable for the activation of indolic defense metabolism

**DOI:** 10.1111/tpj.71130

**Published:** 2026-09-21

**Authors:** Mona Perrar, Marlene Gross, Patrick Petzsch, Karl Köhrer, Jürgen Zeier

**Affiliations:** ^1^ Department of Biology, Institute for Molecular Ecophysiology of Plants Heinrich Heine University Universitätsstraße 1 Düsseldorf D‐40225 Germany; ^2^ Biological and Medical Research Center (BMFZ), Medical Faculty Heinrich Heine University Universitätsstraße 1 Düsseldorf D‐40225 Germany; ^3^ Cluster of Excellence on Plant Sciences (CEPLAS) Heinrich Heine University Universitätsstraße 1 Düsseldorf D‐40225 Germany

**Keywords:** CBP60g, defense priming, defense‐related metabolism, N‐hydroxypipecolic acid, salicylic acid, SARD1, systemic acquired resistance, transcriptional regulation

## Abstract

We investigated the roles of the cognate, redundantly acting transcription factors SARD1 and CPB60g in the metabolic reprogramming of leaves of *Arabidopsis thaliana* suffering from bacterial infection or UV light exposure. Comprehensive metabolite analyses reveal that SARD1/CBP60g exert distinct influences on major stress‐inducible metabolic pathways. The SARD1/CBP60g transcriptional node decisively boosts the biosynthesis of the systemic acquired resistance (SAR)‐inducing metabolites N‐hydroxypipecolic acid (NHP) and salicylic acid (SA), as well as their metabolism to major glucose conjugates. Additionally, it significantly promotes the biotic stress‐induced accumulation of aromatic and branched‐chain amino acids, and the generation of the antioxidant γ‐tocopherol. By contrast, SARD1/CBP60g does not impact or yet exerts negative influence on the stress‐induced biosynthesis of indolic defense compounds, including the phytoalexin camalexin, accumulation of Lys, Ser and Gly, and sterol desaturation. Significantly, we show that SARD1/CBP60g also acts downstream of NHP and SA in immune signaling, indicating a critical function of this transcriptional node in an NHP‐ and SA‐driven immune amplification relay operating in SAR. RNA‐sequencing analysis highlight that the transcriptional response to NHP is separable into two gene groups with different regulatory characteristics. One group consists of strongly SARD1/CBP60g‐promoted genes that invariably contain NHP and SA‐pathway genes and is enriched in SA‐inducible genes. Genes of the other group, which overrepresents H_2_O_2_‐inducible genes and concentrates genes of indolic metabolism, are not promoted by SARD1/CBP60g. Interestingly, SARD1/CBP60g oppositely affect the NHP‐mediated priming of pathogen‐induced SA and camalexin biosynthesis as well, illustrating that plants realize priming of distinct defenses by different mechanisms.

## Significance Statement

The SARD1/CBP60g transcriptional node distinctly influences different stress‐inducible plant metabolic pathways. It both functions in the biosynthesis and in downstream signaling of the systemic acquired resistance inducer N‐hydroxypipecolic acid but does not promote indolic defense metabolism.

## INTRODUCTION

As part of their inducible defense arsenal, plants activate diverse biochemical pathways that result in the synthesis, accumulation, and further metabolization of small immune‐relevant metabolites in response to pathogen attack. These substances include hormone‐ and signaling‐active metabolites, antimicrobial phytoalexins, redox‐active compounds, and various primary metabolites (Ahuja et al., 2012; Stahl et al., 2019; Zeier, 2021).

For example, leaves of the model plant *Arabidopsis thaliana* (Arabidopsis) that interact with pathogenic *Pseudomonas syringae* bacteria accumulate the aromatic amino acids (AAAs) Phe, Tyr, and Trp, the branched‐chain amino acids (BCAAs) Val, Leu, and Ile, and Lys (Návarová et al., 2012; Zeier, 2013). These canonical amino acids may serve as biosynthetic precursors for various immune‐related metabolites. Antioxidant tocopherols are synthesized from Tyr in *P. syringae*‐inoculated leaves and contribute to plant basal immunity by attenuating the oxidation of unsaturated membrane lipids (Stahl et al., 2019). Furthermore, Arabidopsis induces a comprehensive Trp‐derived defensive metabolism upon microbial attack that culminates in the accumulation of the phytoalexin camalexin and other indolic metabolites with potential antimicrobial or signaling function (Bednarek et al., 2009; Glawischnig, 2007; Rajniak et al., 2015; Stahl et al., 2016). These indolics are also generated upon abiotic stress and chemical treatments that trigger production of reactive oxygen species (ROS), suggesting that stress‐related ROS function as a trigger for their production (Glawischnig, 2007; Müller et al., 2019).

Another major branch of pathogen‐inducible metabolism in plants starts from L‐Lys and evokes accumulation of N‐hydroxypipecolic acid (NHP), an immune‐regulatory metabolite that induces a state of broad‐spectrum immunity in plants known as systemic acquired resistance (SAR) (Hartmann et al., 2018; Schnake et al., 2020). NHP is generated in both the locally attacked and in thereof distant leaves of pathogen‐inoculated Arabidopsis plants by the N‐hydroxylation of the non‐canonical amino acid pipecolic acid (Pip) via FLAVIN‐DEPENDENT MONOOXYGENASE1 (FMO1) (Chen et al., 2018; Hartmann et al., 2018). Pip as the precursor of the immune‐active NHP also heavily accumulates in the foliage after pathogen attack and is synthesized from L‐Lys via consecutive transamination and reduction steps catalyzed by the aminotransferase AGD2‐LIKE DEFENSE RESPONSE PROTEIN1 (ALD1) and the reductase SAR‐DEFICIENT4 (SARD4), respectively (Ding et al., 2016; Hartmann et al., 2017; Návarová et al., 2012; Zeier, 2021).

The endogenous accumulation of NHP in plants is indispensable for the establishment of pathogen‐induced SAR (Hartmann et al., 2018; Mishina & Zeier, 2006). In addition, exogenous treatment of individual Arabidopsis rosette leaves with physiological doses of NHP is sufficient to trigger NHP transport and SAR in distant leaves of the Arabidopsis leaf rosette (Hartmann et al., 2018; Chen et al., 20 218; Yildiz et al., 2021). Similarly, it was shown that a localized expression of the key NHP biosynthetic genes in tomato leaflets induced NHP accumulation and SAR in distant leaflets (Holmes et al., 2019). These findings indicate an important function for NHP in SAR long‐distance communication (Zeier, 2021), a process in which ROS and extracellular NAD(P) are involved as well (Cao et al., 2024; Li et al., 2023). Accumulating levels of NHP in plants induce the enhanced transcription of a battery of SAR‐related genes in unchallenged leaves, and this transcriptional reprogramming primes plants for a boosted mobilization of metabolic and other responses in the course of a further pathogen attack (Yildiz et al., 2021). Responses that are primed by NHP in SAR‐activated plants include the pathogen‐ or elicitor‐induced accumulation of camalexin, biosynthesis of salicylic acid (SA), expression of different SAR‐related genes, and the hypersensitive cell death response (HR) (Bernsdorff et al., 2016; Chen et al., 2018; Foret et al., 2025; Hartmann et al., 2018; Löwe et al., 2023).

SA is a defense hormone with important functions in plant basal resistance to compatible pathogens, effector‐triggered immunity to incompatible, HR‐inducing pathogens, and SAR (Klessig et al., 2018). Previous work indicates that SA exhibits an amplifier function for NHP‐triggered responses that is required for an effective activation of SAR (Bernsdorff et al., 2016; Hartmann et al., 2018; Yildiz et al., 2021). Basically, two metabolic routes for the biosynthesis of SA in plants have been identified—the isochorismate synthase (ICS) and the phenylalanine ammonia lyase (PAL) pathways. Whereas tobacco, tomato, cotton, rice, and wheat primarily use the PAL pathway for SA production, Arabidopsis employs the ICS pathway for the stress‐inducible biosynthesis of SA (Liu et al., 2025; Nawrath & Métraux, 1999; Scholten et al., 2024; Wang et al., 2025; Wildermuth et al., 2001; Zhu et al., 2025). In the latter, the shikimate pathway intermediate chorismate is converted into isochorismate by plastid‐resident ISOCHORISMATE SYNTHASE1 (ICS1), which is exported into the cytosol by the presumed isochorismate transporter ENHANCED DISEASE SUSCEPTIBILITY5 (EDS5) and then converted to SA via conjugation to L‐Glu by the amido synthetase avrPphB SUSCEPTIBLE3 (PBS3) and subsequent aromatization (Rekhter et al., 2019; Torrens‐Spence et al., 2019).

Notably, plants have developed a biochemical mechanism to inactivate the two key SAR‐inducing metabolites NHP and SA at once via glycosylation by the same, pathogen‐inducible uridine diphosphate (UDP)‐dependent glycosyltransferase, UGT76B1, to generate the respective inactive β‐glucosides, NHP‐N‐O‐glucoside (NHPG) and SA‐O‐glucoside (SAG) (Bauer et al., 2021; Cai et al., 2021). On the one hand, this terminates defense signal transduction in SAR‐induced plants, and on the other hand, ensures a reduced basal immune level in non‐attacked plants to optimally balance growth and defense (Xu et al., 2025; Zeier, 2021). For SA, another prominent catabolic inactivation route exists via hydroxylation and glycosylation reactions to form glycosides of 2,3‐ and 2,5‐dihydroxybenzoic acids (DHBAs) (Zhang et al., 2013, 2017).

The pathogen‐inducible NHP and SA metabolic pathways in Arabidopsis and the immune‐stimulating action of NHP and SA, respectively, are controlled by partially overlapping regulatory and downstream acting elements (Zeier, 2021). For example, both the biosynthetic pathways of NHP and SA are positively regulated by the immune‐regulatory proteins EDS1 and PHYTOALEXIN‐DEFICIENT4 (PAD4), as well as the transcription factors (TFs) TGA1 and TGA4 (Cui et al., 2017; Hartmann et al., 2018; Sun et al., 2018; Yildiz et al., 2023). Conversely, CAMTA and NAC factors negatively regulate both biosynthetic pathways (Cai et al., 2024; Kim et al., 2020; Sun et al., 2020). Moreover, the two pathogen‐responsive Arabidopsis TFs SARD1 and CALMODULIN‐BINDING PROTEIN 60‐LIKE G (CBP60g) redundantly promote expression of SA‐biosynthetic genes, accumulation of free and glycosidically bound SA, and SAR activation (Wang et al., 2011; Zhang et al., 2010). SARD1 and CBP60g also positively regulate transcript accumulation of the NHP biosynthetic genes *ALD1* and *FMO1* and accumulation of the NHP precursor Pip (Nair et al., 2021; Sun et al., 2015, 2018).

The transcriptional co‐activator and bona fide SA‐receptor NONEXPRESSOR OF PR GENES1 (NPR1) and the clade II TGA TFs TGA2, TGA5, and TGA6 are critical components downstream of NHP in SAR activation, since they are required to transduce elevated NHP levels into SAR‐related gene expression and elevated resistance (Yildiz et al., 2021, 2023). Interestingly, NHP increases transcript levels of many of the above‐mentioned regulatory components, including SARD1, CBP60g, NPR1, and TGA factors (Bernsdorff et al., 2016; Yildiz et al., 2023), suggesting a strong relevance of feedback amplification mechanisms for SAR establishment (Zeier, 2021).

In the current study, we first examined whether the TFs SARD1 and CBP60g would function downstream of NHP in SAR establishment. We show that SARD1 and CBP60g regulate both NHP‐induced SAR and a specific subset of the NHP‐triggered transcriptional response in a largely redundant manner. Strikingly, while SARD1/CBP60g tightly control the NHP‐induced accumulation of transcripts of SA‐ and NHP‐related metabolic genes, they are not required for the NHP‐activated transcription of genes involved in indolic defense metabolism. More broadly, we identify two sets of NHP‐inducible genes with opposed regulation by SARD1/CBP60g that are differentially enriched in SA‐ and H_2_O_2_‐inducible genes and specific TF binding elements. Furthermore, detailed metabolite analyses directly reveal that SARD1/CBP60g fulfill a central function in the (a)biotic stress‐induced biosynthesis and metabolism of both SA and NHP and stimulate accumulation of aromatic and BCAAs as well as accumulation of γ‐tocopherol. By contrast, SARD1/CBP60g are dispensable for the accumulation of camalexin and other indolic metabolites. Consistent with this specific function in defense‐related metabolism, SARD1/CBP60g proved required for the NHP‐induced priming of pathogen‐triggered SA biosynthesis but dispensable for the NHP‐mediated priming of camalexin accumulation. Our data suggest that SARD1/CBP60g play a critical role in a previously proposed NHP‐related feedback amplification mechanism associated with SAR.

## RESULTS

### The transcription factors SARD1 and CBP60g act downstream of NHP in SAR induction and transduce diverse SAR stimuli in a redundant manner

**Figure 1:**
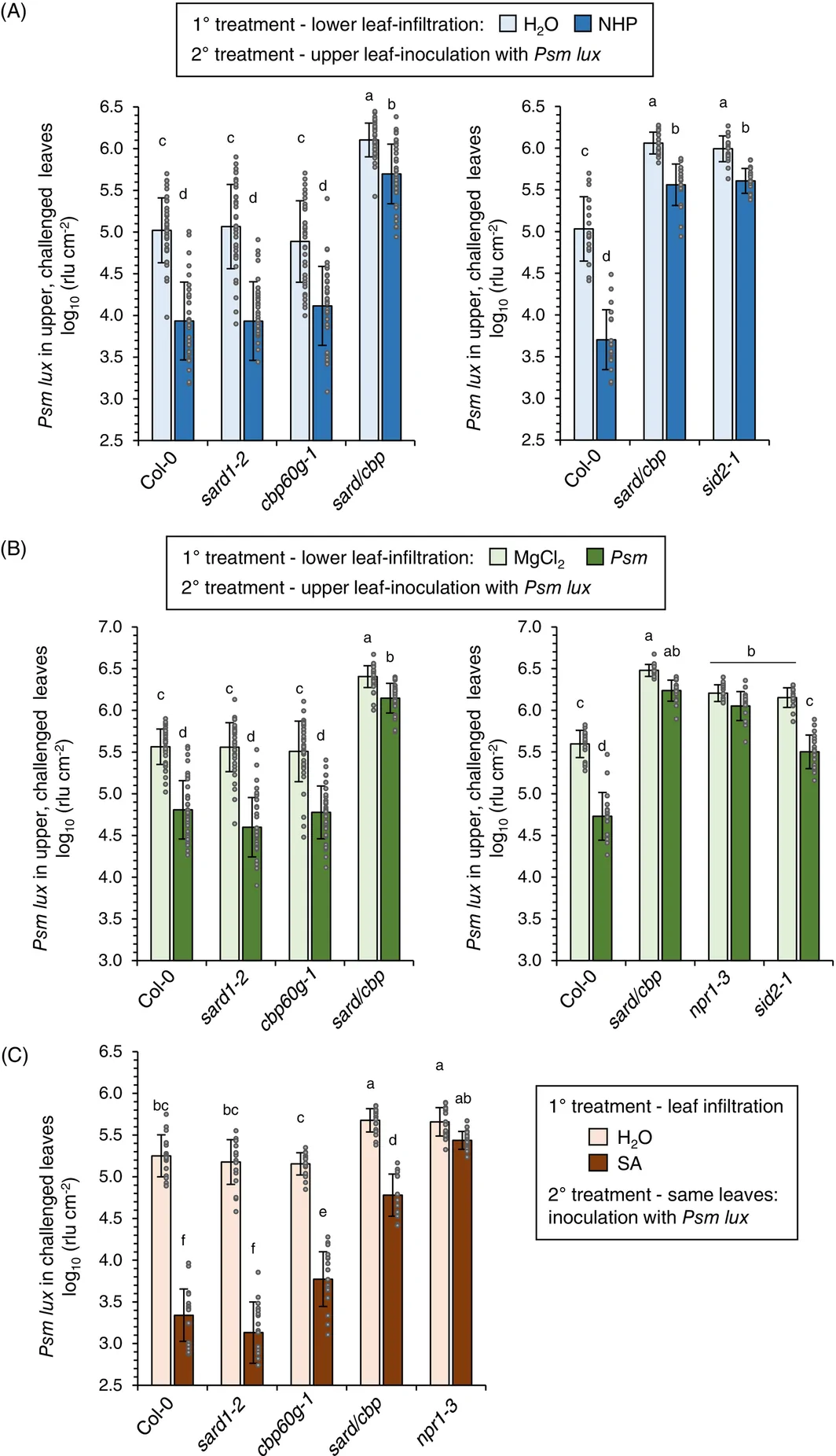
Requirement and redundant action of the transcription factors SARD1 and CBP60g in the establishment of Arabidopsis (systemic) acquired resistance evoked by exogenous N‐hydroxypipecolic acid (NHP) and salicylic acid (SA) treatments, and by localized inoculation with *P. syringae*. (A) Systemic acquired resistance (SAR) establishment by exogenous NHP treatment is similar to wild type in the *sard1*‐*2* (*sard1*) and *cbp60g*‐*1* (*cbp60g*) single mutants, but compromised in the *sard1*‐*2*/*cbp60g*‐*1* (*sard/cbp*) double mutant and in the SA‐biosynthetic mutant *sid2*‐*1* (*sid2*). Plants were syringe‐infiltrated in three lower leaves with either 1 mM NHP or with H_2_O (1° treatment), and 1 day later, three upper leaves were inoculated with *Psm lux* (OD 0.001). Bacterial numbers in leaves were assessed by quantifying the bacterial bioluminescence at 2.5 days post‐inoculation. Bars show the mean ± SD of log_10_‐transformed relative light units (rlu) per cm^2^ leaf area of 34–36 leaf replicates (left) and 17–18 replicates (right), respectively. Gray circles represent individual data points of leaf replicates. Different letters indicate significant differences (*P* < 0.05, ANOVA and post hoc Tukey HSD test). Similar tendencies were observed in three independent experiments. (B) SARD1 and CBP60g mediate the induction of *P. syringae*‐triggered SAR in a redundant manner. The degree of biologically induced SAR in the Arabidopsis lines under investigation was assessed by comparing the outcome of a bacterial challenge of plants in upper leaves with *Psm lux* (OD 0.001) 2 days after they had been treated in lower leaves with *Psm* (OD 0.005; for SAR induction) or with a mock‐solution (10 mM MgCl_2_; uninduced plants). The numbers of *Psm lux* in the challenged leaves were assessed by luminescence measurements at 2.5 days post‐inoculation and expressed as log_10_‐transformed rlu cm^−2^. Bars indicate the mean ± SD of 35–36 leaf replicates (left) and 17–18 replicates (right), respectively. Other details as described in (A). Similar tendencies were observed in three independent experiments. (C) Local acquired resistance in response to exogenous SA is attenuated but not fully abrogated in the *sard/cbp* double mutant. To assess SA‐induced resistance, three leaves of a given plant were pre‐infiltrated with either 0.5 mM SA or with water. Four hours later, the same leaves were challenge‐inoculated with *Psm lux* (OD 0.001), bacterial numbers quantified at 2.5 dpi and expressed as log_10_‐transformed rlu per cm^2^ leaf area. The means ± SD of 17–18 leaf sample values are shown. Other details as described in (A). Similar tendencies were observed in two independent experiments.

To investigate whether SARD1 and CBP60g function downstream of NHP in SAR, we first compared the systemic induction of resistance by exogenously applied NHP in Arabidopsis Col‐0 wild‐type plants, the single knockout mutants *sard1*‐*2* (*sard1*) and *cbp60g*‐*1* (*cbp60g*), and the *sard1*‐*2*/*cbp60g*‐*1* (*sard/cbp*) double mutant (Wang et al., 2011; Zhang et al., 2010). Consistent with previous results (Schnake et al., 2020; Yildiz et al., 2021), infiltration of 1 mM NHP to lower leaves of Col‐0 wild‐type plants induced strong SAR to subsequent infection by compatible, *luxCDABE*‐tagged *P. syringae* pv. *maculicola* (*Psm lux*; Fan et al., 2008; Gruner et al., 2018) in upper leaves (Figure 1A). This NHP‐induced SAR response to *Psm lux* infection was similar in Col‐0, *sard1*, and *cbp60g* plants, but strongly attenuated in *sard/cbp* double mutants. Comparable tendencies in the different lines were observed when SAR in leaves was triggered by supply of a 1 mM NHP solution to the cultivation soil, confirming mechanistic overlap of SAR induction by leaf and soil treatments (Figure S1; Yildiz et al., 2021, 2023). Moreover, basal immunity to bacterial infection of naïve or water‐pre‐infiltrated control plants was greatly reduced in the *sard/cbp* double mutant, while the single mutants showed a wild‐type‐like resistance (Figure 1A; Figure S1). Both NHP‐induced SAR and basal immunity were compromised to a similar degree in *sard/cbp* plants and in the SA‐induction‐deficient line *sid2* (Figure 1A; right graph).

To compare the NHP‐induced SAR responses in the lines under investigation with their capacities of acquiring SAR biologically in response to bacterial attack, we inoculated lower leaves of plants with *P. syringae pv. maculicola* (*Psm*) for SAR induction, challenged upper leaves 2 days later with *Psm lux*, and assessed bacterial multiplication in the upper leaves another 2.5 days later (Návarová et al., 2012). Mock‐treated plants that were infiltrated in lower leaves with a MgCl_2_ solution before *Psm lux* challenge thereby served as controls (Figure 1B). Whereas Col‐0, *sard1*, and *cbp60g* plants developed strong SAR in the distant leaves of locally *Psm*‐pre‐inoculated plants, the *sard/cbp* double mutant was only able to induce a weak biological SAR response (Zhang et al., 2010), which was quantitatively in between the responses of the SA‐ and NHP‐insensitive *npr1* line (no SAR; Yildiz et al., 2021) and the *sid2* mutant (modest SAR; Bernsdorff et al., 2016) (Figure 1B).

Plant leaves also locally acquire resistance after treatment with exogenous SA (Delaney et al., 1995). To examine whether differences in SA‐induced resistance existed between the lines under investigation, we infiltrated the leaves of given plants with 0.5 mM SA and challenged the same leaves 4 h later with *Psm lux* (Figure 1C; Yildiz et al., 2023). The SA treatment evoked a strong resistance induction in the leaves for Col‐0, *sard1*, and *cpb60g*, with the latter line reacting modestly lower to SA than the two former lines (Figure 1C). By comparison, the *sard/cbp* double mutant exhibited a significantly lower resistance increase in response to exogenous SA than each of the single mutants or the wild type, but still responded stronger than *npr1* (Cao et al., 1997; Yildiz et al., 2023), which proved to be virtually SA‐insensitive (Figure 1C).

Together, these results corroborate previous findings that SARD1 and CBP60g are important immune components that act in a redundant manner in the induction of SAR (Wang et al., 2011; Zhang et al., 2010; Figure 1B). Significantly, they establish that SARD1 and CBP60g redundantly function downstream of NHP and SA in the resistance pathways leading to SAR. Both NHP‐ and SA‐triggered induction of resistance is strongly attenuated by a simultaneous dysfunction of both TFs but proceeds at normal strength when one of the factors is functional (Figure 1A,C).

### SARD1 and CBP60g positively regulate a subcategory of the transcriptional response to NHP that involves expression of SA‐ and NHP‐metabolic genes

SAR triggered by a local bacterial inoculation of Arabidopsis Col‐0 leaves is associated with a massive transcriptional response in distant, systemic leaves for which the endogenous accumulation of NHP is essential (Bernsdorff et al., 2016; Gruner et al., 2013; Hartmann et al., 2018; Mishina & Zeier, 2006). Remarkably, the exogenous application of NHP to Col‐0 plants via soil (or leaf) treatments evokes a transcriptional reprogramming in the leaves that is highly similar to the response observed in pathogen‐induced, biological SAR (Yildiz et al., 2021). Our previous studies have established that the NHP‐triggered transcriptional response is significantly attenuated in SA‐deficient *sid2* plants and virtually absent in both *npr1* and *tga2/tga5/tga6* triple mutants, which suffer from simultaneous loss of the three TGA family TFs TGA2, TGA5, and TGA6 (Yildiz et al., 2021, 2023).

**Figure 2:**
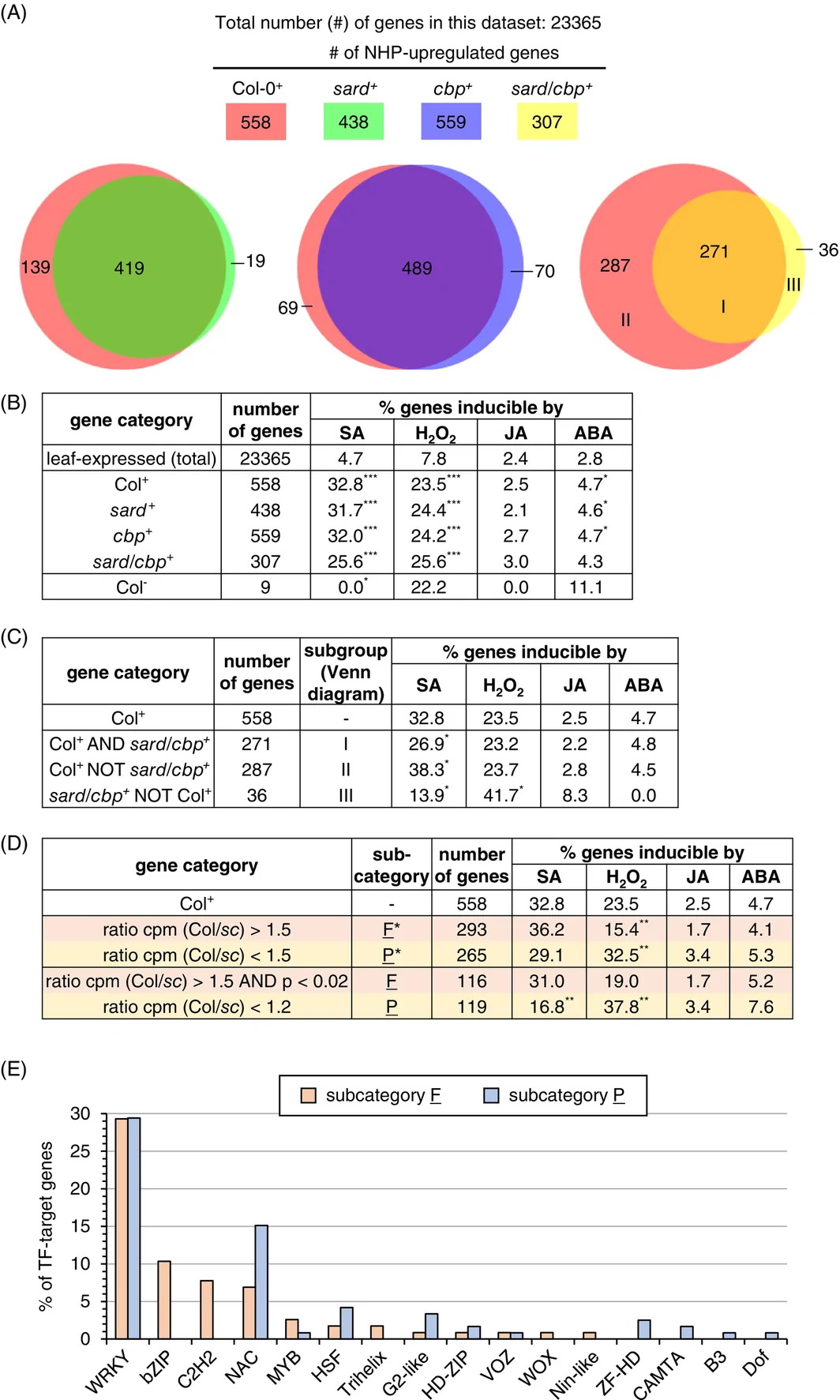
SARD1 and CBP60g amplify a particular subset of N‐hydroxypipecolic acid (NHP)‐inducible genes. To compare the transcriptional response of Col‐0 wild‐type, *sard1*, *cpb60g*, and *sard/cbp* plants to NHP at the whole‐genome level, six plants of each genotype were watered with 10 ml of 1 mM NHP, and six other plants were watered with 10 ml of H_2_O as a control treatment. RNA extracts from the 18 harvested leaves of the same genotype/treatment combination were pooled to one sample which was used for RNA‐sequencing analysis. Together, three independent experiments with identical setups were performed so that 24 samples consisting of three independent samples of each genotype/treatment combination were analyzed by RNA‐seq (Figure S2). (A) Out of 27 655 RNA‐seq‐covered genes, 4290 genes showed counts per million (cpm) values = 0 for all of the 24 examined leaf samples and were excluded from further analyses. Top: Numbers of NHP‐upregulated genes out of the 23 365 expressed genes in the leaves of Col‐0, *sard1*, *cpb60g*, and *sard/cbp* plants (‘Col^+^’, ‘*sard* ^+^’, ‘*cbp* ^+^’ and ‘*sard/cbp* ^+^’ genes). Genes were considered as upregulated (down‐regulated) by NHP when false discovery rate (FDR)‐adjusted *P*‐values from comparison of NHP‐ and H_2_O‐samples were ≤0.005 and NHP/H_2_O‐ratios of mean cpm values were ≥3.0 (≤0.33) (Data S1). Bottom: Venn diagrams illustrating overlap between *sard* ^+^‐, *cbp* ^+^‐ or *sard/cbp* ^+^‐genes with Col^+^ genes, respectively. (B) Percentages of salicylic acid (SA)‐inducible genes (Thibaud‐Nissen et al., 2006; 1105 genes in total), H_2_O_2_‐inducible genes (Davletova et al., 2005; 1856 genes in total), jasmonic acid (JA)‐inducible genes (Goda et al., 2008; 576 in total), and abscisic acid (ABA)‐inducible genes (Goda et al., 2008; 675 genes in total) in the Col^+^‐, *sard* ^+^‐, *cbp* ^+^‐, *sard/cbp* ^+^‐, and Col^−^ (down‐regulated in Col‐0 by NHP)‐ gene groups. To determine whether the indicated gene categories were significantly enriched or depleted in hormone‐inducible genes compared to the total group of expressed genes, Fisher's exact test was used (*P < 0.05, **P < 0.001, ***P < 10^−6^). (C) Subgroups of genes with different NHP‐inducibility in Col‐0 and *sard/cbp* plants (see also right Venn diagram depicted in (A)). Subgroup I: genes significantly induced (FDR <0.005; fold change >3) in both Col‐0 and *sard/cbp*; Subgroup II: genes significantly induced in Col‐0 but not in *sard/cbp*; Subgroup III: genes significantly induced in *sard/cbp* but not in Col‐0. The percentages of stress‐hormone‐inducible genes in these subgroups are compared. (D) Subcategories of the Col^+^ genes whose NHP‐induced expression either show or lack pronounced positive regulation by SARD1/CPB60g. In a first step, genes with Col‐0‐to‐*sard/cbp*‐ratios of cpm values of NHP‐treatment samples of >1.5 were grouped together (subcategory F* containing FMO1; positively regulated by SARD1/CPB60g), while such with ratios ≤1.5 were combined (subcategory P* containing PAD3; no pronounced positive regulation by SARD1/CPB60g). The stringency of these regulation criteria was further increased. The F* group was reduced to a subcategory designated as F by only considering genes with significant differences (*P* < 0.02) between the NHP‐Col‐0 and the NHP‐*sard/cbp*‐samples. In addition, the P* group was reduced to the subcategory P by excluding genes with Col‐0‐to‐sard/cbp‐ratios of cpm values of NHP‐treatment samples >1.2. While the subcategory F, by definition, unexceptionally consisted of genes with higher NHP‐inducibility in Col‐0 than in *sard/cbp* (*sc*), subcategory P genes contained genes with similar NHP‐inducibility in both genotypes or, less frequently, genes more pronouncedly expressed in *sard/cbp* than in Col‐0 (Figure 3, Figure S2, Data S1). The percentages of stress‐hormone‐inducible genes in these subcategories were determined as described in (C). (E) To identify enriched regulatory elements in the genes of the F‐ and P subcategories, TF‐motif differential enrichment analysis of the F‐ and P‐ subcategories of NHP‐responsive genes using the Plant Transcriptional Regulatory Map database (PTRMD) (https://plantregmap.gao‐lab.org/tf_enrichment.php; Tian et al., 2020) was performed. The bars depict the percentages of target genes of the indicated transcription factors in the F‐ and P‐ subcategory gene lists (abbreviations of TF classes: bZIP, basic leucine zipper; NAC, NO APICAL MERISTEM/ATAF/CUP SHAPED COTYLEDON; C2H2, Cysteine2/Histidine2‐type of zinc finger; MYB, myeloblastosis; HSF, heat shock factor; G2, Golden2‐like; HD‐ZIP, homeodomain‐leucine zipper; VOZ, VASCULAR PLANT ONE‐ZINC FINGER PROTEIN; WOX, WUSCHEL‐related homeobox; Nin‐like, nodule inception‐like; ZF‐HD, zinc finger‐homeodomain; CAMTA, calmodulin‐binding transcription activator; B3, B3‐domain containing; Dof, DNA binding with one finger).

To directly examine the roles of the TFs SARD1 and CBP60g in the NHP‐triggered transcriptional SAR response, we performed an RNA‐sequencing (RNA‐seq)‐based assessment of changes in gene expression caused by exogenous NHP in the leaves of Col‐0, *sard1*, *cbp60g*, and *sard/cbp* plants (Figure 2). To stay comparable with previous studies, plants were supplied via the soil with either 10 ml of 1 mM NHP as an inducing treatment or with 10 ml of H_2_O as a control treatment (Yildiz et al., 2021, 2023). Leaves of treated plants were harvested 1 day later for transcriptional analysis. As described in Table S1, three independent biological experiments were conducted so that expression values of three biological replicate samples per genotype and treatment could be statistically analyzed (Figure 2).

The RNA‐seq analysis comprised 27 655 Arabidopsis genes, of which 4290 genes did not show expression in any of the examined 24 leaf samples [counts per million (cpm) = 0]. We excluded these genes from further analysis and thus obtained a total number of 23 365 leaf‐expressed genes for detailed evaluation (Figure 2A,B). To define subsets of genes with a robust differential regulation following NHP‐ compared with H_2_O treatment, we determined the genes exhibiting false discovery rate (FDR)‐adjusted *P*‐values ≤0.005 for the NHP‐ and H_2_O‐values of one genotype, and showing treatment‐related fold changes ≥3.0 for the mean of cpm values. Genes with *P*‐values ≤0.005 and NHP/H_2_O‐ratios ≥3.0 (≤0.33) were considered as robustly upregulated (downregulated) in response to NHP and designated as Col^+^ (Col^−^) genes for Col‐0, *sard* ^+^ (*sard* ^*−*^) genes for *sard1*, *cbp* ^+^ (*cbp* ^*−*^) genes for *cbp60g*, and *sard/cbp* ^+^ (*sard/cbp* ^*−*^) genes for *sard/cbp*, respectively (Figure 2A; Data S1).

On the basis of these parameters, 558 NHP‐upregulated Col^+^ genes and nine NHP‐downregulated Col^−^ genes were classified for the wild‐type (Figure 2A,B; Data S1), confirming that gene upregulation is more pronounced than gene downregulation within the transcriptional response to NHP (Yildiz et al., 2021, 2023). Out of the 558 identified Col^+^ genes, 551 (98.7%) also fell into the group of NHP‐upregulated genes in our previous RNA‐seq study (Yildiz et al., 2023), verifying yet again that NHP robustly induces a defined SAR‐associated subset of genes of the Arabidopsis genome (Bernsdorff et al., 2016; Yildiz et al., 2021). To obtain more insights into the expression characteristics of the Col^+^ genes compared with the entirety of 23 365 leaf‐expressed genes, we merged the RNA‐seq data set with lists of genes previously identified as being inducible by the plant stress hormones or signaling compounds SA, jasmonic acid (JA), abscisic acid (ABA) and H_2_O_2_ (Davletova et al., 2005; Goda et al., 2008; Gruner et al., 2013; Thibaud‐Nissen et al., 2006). This analysis confirmed that the group of NHP‐induced Col^+^ genes are strongly enriched in SA‐ and H_2_O_2_‐inducible genes, while ABA‐ and JA‐inducible genes show modest or no significant enrichment, respectively (Figure 2B; Yildiz et al., 2023). By contrast, SA‐inducible genes were significantly depleted (i.e., not present) in the group of the NHP‐downregulated Col^−^ genes (Figure 2B).

The numbers of NHP‐induced genes of the *sard1* and *cbp60g* mutant amounted to 438 and 559 genes, respectively, and both the *sard* ^+^ (95.7%) and *cbp* ^+^ (87.4%) gene groups greatly overlapped with the 558 Col^+^ genes (Figure 2A). Moreover, the genes of both the *cbp* ^+^ and *sard* ^+^ gene groups shared similar characteristics regarding their SA‐, H_2_O_2_‐, and ABA‐inducibility with the Col^+^ gene group (Figure 2B). Thus, the *cbp60g* mutant exhibited wild‐type‐like transcriptional responses to NHP, and *sard1* showed a modestly lower response than the wild type on a quantitative basis. Notably, as observed for the induction of SAR by exogenous NHP (Figure 1A), the transcriptional response to NHP was most strongly affected in *sard/cbp* plants (Figure 2A). Compared with the 558 Col^+^ genes, merely 307 NHP‐upregulated *sard/cbp* ^+^ genes were determined for the double mutant (Figure 2A). Moreover, the percentage of SA‐inducible genes was lower among the *sard/cbp* ^+^ genes (25.6%) than among the Col^+^ genes (32.8%) (Figure 2B), indicating differences in the regulation of *SARD1/CPB60g*‐affected and *SARD1/CPB60g*‐unaffected genes following their induction by NHP.

To better understand the nature and regulatory principles of the genes that are differently and similarly expressed in *sard/cbp* and wild‐type plants in response to NHP, respectively, we classified three distinct gene subgroups (Figure 2A,C): Subgroup I consisted of 271 genes that were, according to our initially defined criteria (FDR <0.005; fold change >3), significantly induced in both Col‐0 and *sard/cbp*, Subgroup II contained the remaining 287 Col^+^ genes that were significantly induced in Col‐0 but not in *sard/cbp*, and Subgroup III consisted of 36 genes significantly induced in the *sard/cbp* double mutant but not in Col‐0 (Figure 2A, right Venn diagram). After quantifying the percentages of hormone‐inducible genes in these subgroups, it emerged that, compared with Col^+^ as a reference group, Subgroup II was significantly enriched in SA‐inducible genes, while Subgroup I and, particularly, Subgroup III, were significantly depleted in SA‐inducible genes (Figure 2C). Thus, the relative number of SA‐inducible genes was higher in the gene group whose members were positively regulated by functional SARD1/CPB60g than in the other two groups that contained genes with full or partial SARD1/CPB60g‐independent expression characteristics. Moreover, the relative number of H_2_O_2_‐inducible genes was markedly enriched in Subgroup III (Figure 2C). Together, our analysis revealed differential regulation of NHP‐induced genes that are expressed in dependence and in (partial) independence of functional SARD1/CPB60g.

To further investigate this issue on a defined quantitative basis, we divided the 558 Col^+^ genes into genes that either exhibit or lack a marked positive regulation by SARD1/CPB60g with respect to their NHP‐inducible expression. For this purpose, we first regrouped the Col^+^ genes into two subcategories, one with Col‐0‐to‐*sard/cbp*‐ratios of cpm values of NHP‐treatment samples of >1.5 (293 genes, involving *FMO1* as a characteristic SARD1/CPB60g‐promoted gene) and another with ratios ≤1.5 (265 genes, involving *PAD3* as a typical SARD1/CPB60g‐independent gene). We therefore named the two gene groups after these two paradigm genes subcategory F* and P*, respectively (Figure 1D). To increase the stringency of the criterion ‘SARD1/CPB60g‐promoted expression’ within the F* group, we only considered genes with significant differences (*P* < 0.02) between the NHP‐Col‐0 and the NHP‐*sard/cbp*‐samples, which resulted in a reduction of this group to 116 genes whose expression exhibited a strong dependence on functional SARD1/CPB60g. This gene group was designated as ‘subcategory F’ (Figure 2D). Moreover, the stringency of the criterion ‘lack of SARD1/CPB60g‐promoted expression’ was increased in the P* group by excluding genes with Col‐0‐to‐*sard/cbp*‐ratios of cpm values >1.2. This resulted in 119 genes with strong NHP‐induced expression in the *sard/cbp* double mutant, and the respective gene group was designated as ‘subcategory P’. Strikingly, compared with the Col^+^ and the subcategory F genes, the subcategory P genes were substantially depleted in SA‐inducible genes and strongly enriched in H_2_O_2_‐inducible genes (Figure 2D). This analysis corroborated the above assumption that NHP‐inducible genes whose expression is not promoted by SARD1/CPB60g are generally more likely to be H_2_O_2_‐responsive but less likely to show SA‐inducibility than genes that are positively regulated by SARD1/CPB60g (Figure 2D).

**Figure 3:**
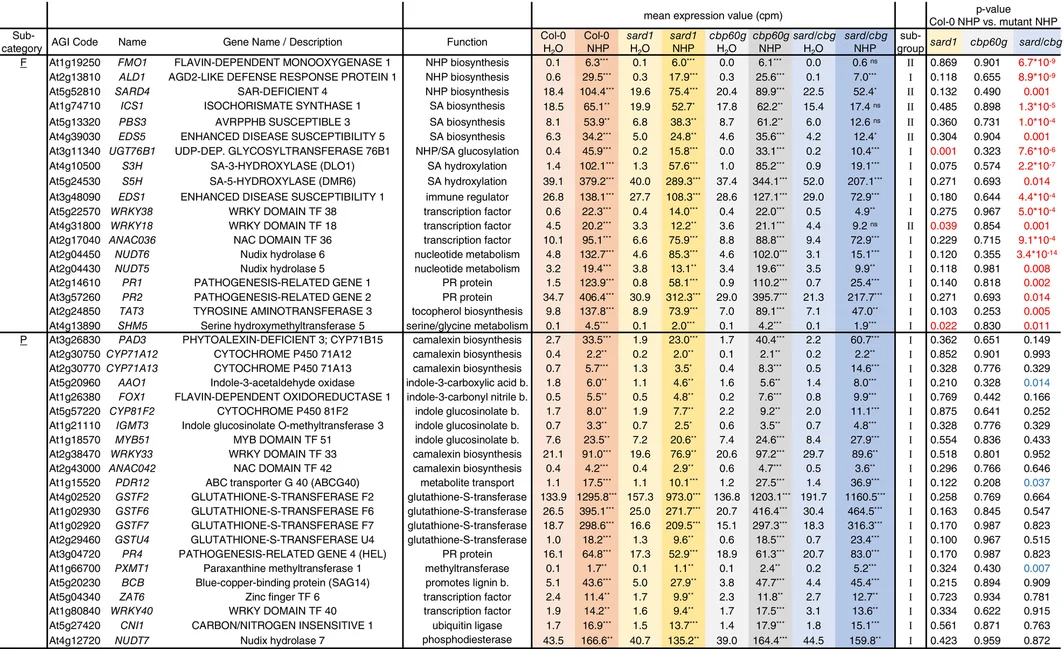
SARD1 and CBP60g mediate expression of N‐hydroxypipecolic acid (NHP)‐inducible genes involved in particular metabolic defense pathways. Selected genes of subcategories F (top) and P (bottom) of the Col^+^ category of NHP‐inducible genes are listed, and their reported biological functions are given. The complete gene lists are provided in Data S1. Mean expression values (cpm) of H_2_O‐control‐ (lighter backgrounds) and NHP‐treatment‐samples (darker backgrounds) of Col‐0 (red), *sard1* (yellow), *cpb60g* (gray), and *sard/cbp* (blue) are given. Asterisks behind the cpm values of NHP‐treatment‐samples indicate significant differences between NHP‐ and H_2_O‐treatments (***: FDR < 10–5, **: FDR <0.005, *: FDR <0.05, no sign: FDR ≥0.05). On the right, the subgroup classification of the genes (I, II or III; Figure 2A,C) are listed, and *P*‐values from the comparisons of Col‐0 and mutant NHP‐treatment‐samples are given. Red (blue) coloration of *P*‐values indicates weaker (stronger) expression in the mutant than in Col‐0. Black coloration of *P*‐values indicates no significant change between the mutant and the wild‐type.

When analyzing the nature of the F‐ and P‐subcategory genes, we observed a surprisingly clear tendency with respect to the distribution of genes in distinct metabolic defense pathways (Figure 3; Figure S2): On the one hand, genes involved in the biosynthesis of NHP (*ALD1*, *SARD4*, and *FMO1*) and SA (*ICS1*, *EDS5*, and *PBS3*), in the metabolic conversion of these two SAR‐regulatory compounds (*UGT76B1*, *S3H*, and *S5H*), in the regulation of the respective metabolic pathways (*EDS1*), and genes that characteristically act downstream in SA‐ and NHP‐signaling (*PR1*) belonged to subcategory F of SARD1/CPB60g‐promoted genes. They were either expressed in full dependence of functional SARD1/CPB60g and thus belonged to the Subgroup II genes (Figure 2A,C; e.g., *FMO1*, *ICS1*, and *PBS3*) or still showed statistically significant (but markedly reduced) expression in the *sard/cbp* double mutant and therefore were among to the Subgroup I genes (Figure 2A,C; e.g., *ALD1*, *UGT76B1*, and *PR1*) (Figure 3; Figure S2). A small part of F‐subcategory genes also showed a significantly lower NHP‐induced expression in the *sard1* single mutant than in the wild type (e.g., *UGT76B1* and *WRKY18*; Figure 3).

On the other hand, NHP‐inducible genes involved in the biosynthesis of the Trp‐derived phytoalexin camalexin (*PAD3*, *CYP71A12*, and *CYP71A13*) and other indolic metabolites such as indole‐3‐carboxylic acid (ICA) (*AAO1*), indole‐3‐carbonitrile (ICN) (*FOX1*), indolic glucosinolates (*CYP81F2*, *IGMT3*), and TFs participating in the regulation of indolic metabolite pathways (*MYB51*, *WRKY33*, and *ANAC042*) belonged to the P subcategory. Moreover, a series of highly expressed and NHP‐upregulated glutathione‐S‐transferase genes (*GSTF2*, *GSTF6*, *GSTF7*, and *GSTU4*) and of H_2_O_2_‐inducible marker genes (*BCB* and *ZAT6*) were also among the subcategory P genes. Because of their strongly NHP‐induced expression in *sard/cbp*, the subcategory P genes all belonged to the Subgroup I genes. Many of the P genes also showed a tendentially (e.g., *CYP71A13* and *PAD3*) or, in some cases, a significantly higher expression in *sard/cbp* than in Col‐0 plants upon NHP induction (e.g., *AAO1*, *PDR12* and *PXMT1*). The latter feature was obviously also true for the Subgroup III genes that were significantly induced in *sard/cbp* but not in Col‐0 (Figures 2A,C and 3; Figure S2; Data S1).

We further reasoned that the observed distinct expression features of F‐ and P‐subcategory genes might be associated with differences in abundance of specific regulatory elements in the (promoter) sequences of these genes. To identify enriched regulatory elements, we performed TF‐motif differential enrichment analysis of the F‐ and P subcategories of NHP‐responsive genes using the Plant Transcriptional Regulatory Map database (PTRMD) (Tian et al., 2020). The analysis revealed that both the F‐ and P‐subcategory (and therefore the whole Col^+^ gene group) were strongly enriched in WRKY TF target motifs (Figure 2E). In addition, several regulatory elements were differentially enriched in both subcategories. While regulatory elements targeted by basic bZIP‐ and C2H2‐type of zinc finger (C2H2) TFs were only enriched in the F‐subcategory, regulatory sequences targeted by NAC‐, ZF‐HD, and CAMTA‐TFs were more strongly enriched among the P‐ than the F‐subcategory genes, confirming differences in the abundance of particular regulatory elements in both gene groups (Figure 2E). We additionally performed a motif analysis in the promoter regions of the F and P genes using the Arabidopsis Information Resource (TAIR) motif finder tool (Table S3). This analysis compared the abundance of six base sequence motifs in the genome region 1000 bp upstream of the genes of the F and P subcategories to their abundance across all genes in the genome. The 18 and 14 hexamer motifs were found to be specifically enriched in the F and P subcategories, respectively, while four motifs proved enriched in both of the subcategories (Fisher test, *P* < 0.005). Remarkably, the two most strongly enriched gene motifs in the F subcategory were the C‐box motif GACGTC and the sequence TGACGT, which are both preferentially targeted by bZIP TFs that include TGA factors as a subgroup (Izawa et al., 1993; Jakoby et al., 2002; Franco‐Zorrilla et al., 2014). In addition, the W‐box and WT‐box motifs TTGACT and GACTTT were among the two hexamers enriched in both the F and P subcategories (Table S3B; Song et al., 2023). Moreover, several other hexamers enriched in one or both of the two subgroups contained TGAC or the ACGT sequences that are core parts of the recognition elements of WRKY, TGA and bZIP TFs, respectively (Table S3).

In summary, corroborating the above‐described resistance assays (Figure 1A), our RNA‐seq analysis showed that SARD1 and CBP60g act downstream of NHP in the immune pathway leading to SAR (Figures 1, 2, 3; Figures S1 and S2). More precisely, our transcriptome analyses highlight that the two TFs adopt specific functions in NHP‐inducible gene expression that are associated with distinct roles in the mediation of metabolic defense pathways. Predominantly in a redundant manner, SARD1 and CBP60g boost the NHP‐induced expression of biosynthetic, metabolic, regulatory, and downstream genes of the SAR‐associated NHP and SA immune pathways. The respective SARD1/CBP60g‐regulated gene group (subcategory F) is enriched in SA‐inducible genes. By contrast, SARD1/CBP60g are not involved or even negatively affect the expression of genes of a second gene subcategory (P) that is enriched in H_2_O_2_‐inducible genes and specifically comprises genes associated with indolic defense metabolism. The differences in gene regulation are reflected by distinct distribution of defined regulatory elements in the two gene subcategories.

### SARD1 and CBP60g redundantly regulate the stress‐induced biosynthesis of NHP, SA and their glucose conjugates but are dispensable for the accumulation of camalexin and other indolic metabolites

**Figure 4:**
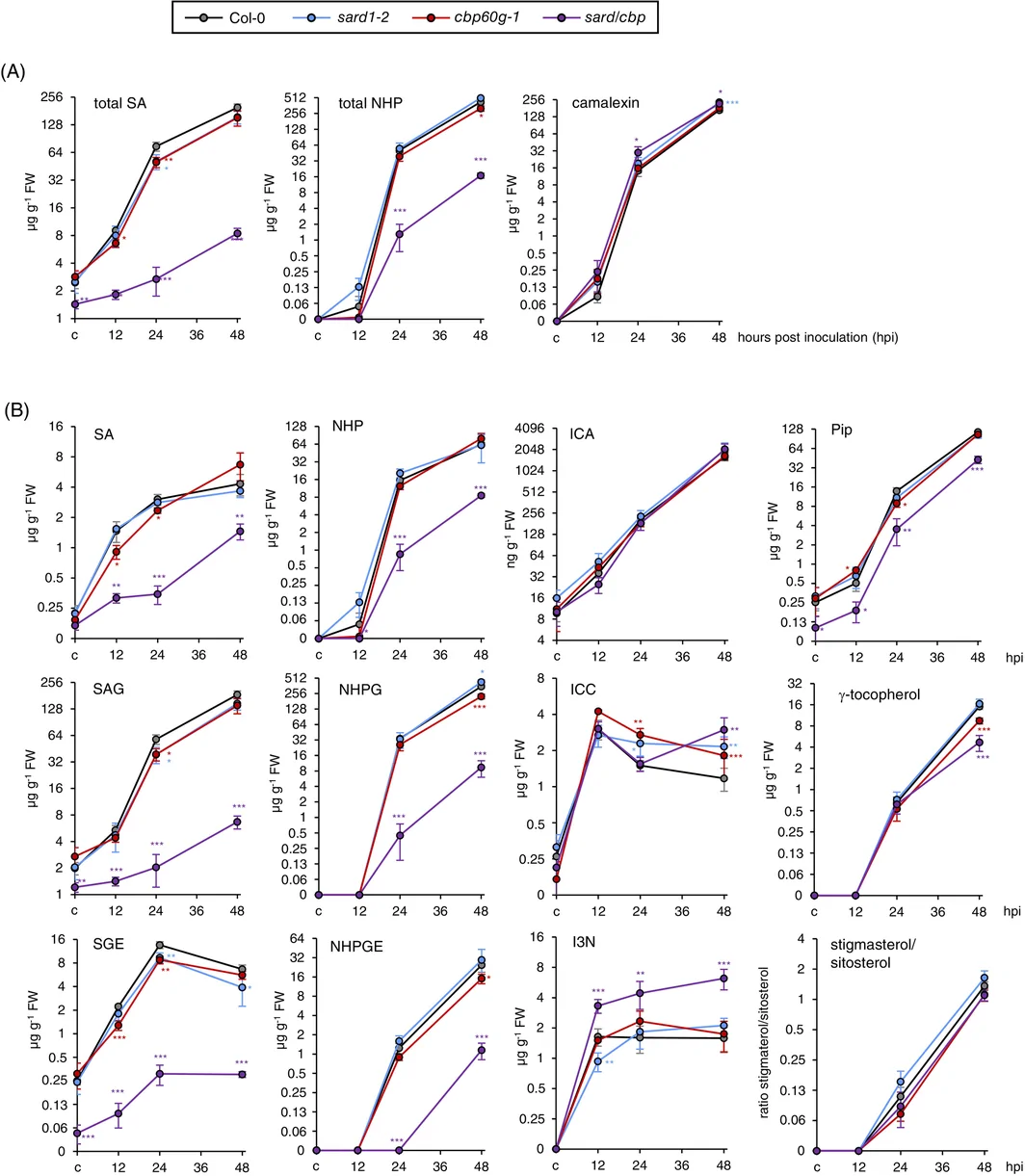
SARD1 and CBP60g mediate the *P. syringae*‐triggered accumulation of salicylic acid (SA), NHP, and the respective glucose conjugates in a redundant manner, but are not required for the inducible biosynthesis of indolic metabolites. (A, B) Time course analysis of metabolite levels in *Psm*‐inoculated leaves of Col‐0 (gray), *sard1* (blue), *cpb60g* (red), and *sard/cbp* (purple) at 12, 24, and 48 hours post‐inoculation (hpi) compared with the levels in non‐inoculated, mock‐infiltrated leaves harvested at 12 h post‐treatment (c). Three leaves of a given plant were *Psm*‐inoculated (OD 0.005) or mock‐treated (10 mM MgCl_2_‐infiltrated), and combined for one sample replicate. The circles indicate the means (± SD) of three to five biological replicates. Asterisks next to the circles denote significant differences between mutant and Col‐0 values (***: *P* < 0.001, **: *P* < 0.01, *: *P* < 0.05; two‐tailed *t*‐test). Metabolite levels are generally given in μg substance per g leaf fresh weight (FW), except for indole‐3‐carboxylic acid (given in ng g^−1^ FW) and stigmasterol (expressed as ratio stigmasterol/β‐sitosterol). To better illustrate low and high metabolite levels in one diagram, most of the y‐axes were log_2_‐transformed. The levels of most of the metabolites were determined by GC–MS analysis, except for indole‐3‐carbaldehyde and indole‐3‐carbonitrile, which were analyzed by LC–MS. (A) Levels of total SA (calculated as the sum of SA, SAG, and SGE), total NHP (total NHP; calculated as the sum of NHP, NHPG, and NHPGE), and camalexin. (B) Levels of SA, SA‐β‐glucoside (SAG), SA glucose ester (SGE), N‐hydroxypipecolic acid (NHP), NHP‐*N*‐*OH*‐glucoside (NHPG), NHP glucose ester (NHPGE), indole‐3‐carboxylic acid (ICA), indole‐3‐carbaldehyde (ICC), indole‐3‐carbonitrile (I3N), pipecolic acid (Pip), γ‐tocopherol, and stigmasterol (relative to β‐sitosterol). Two independent experiments were conducted that yielded similar tendencies.

**Figure 5:**
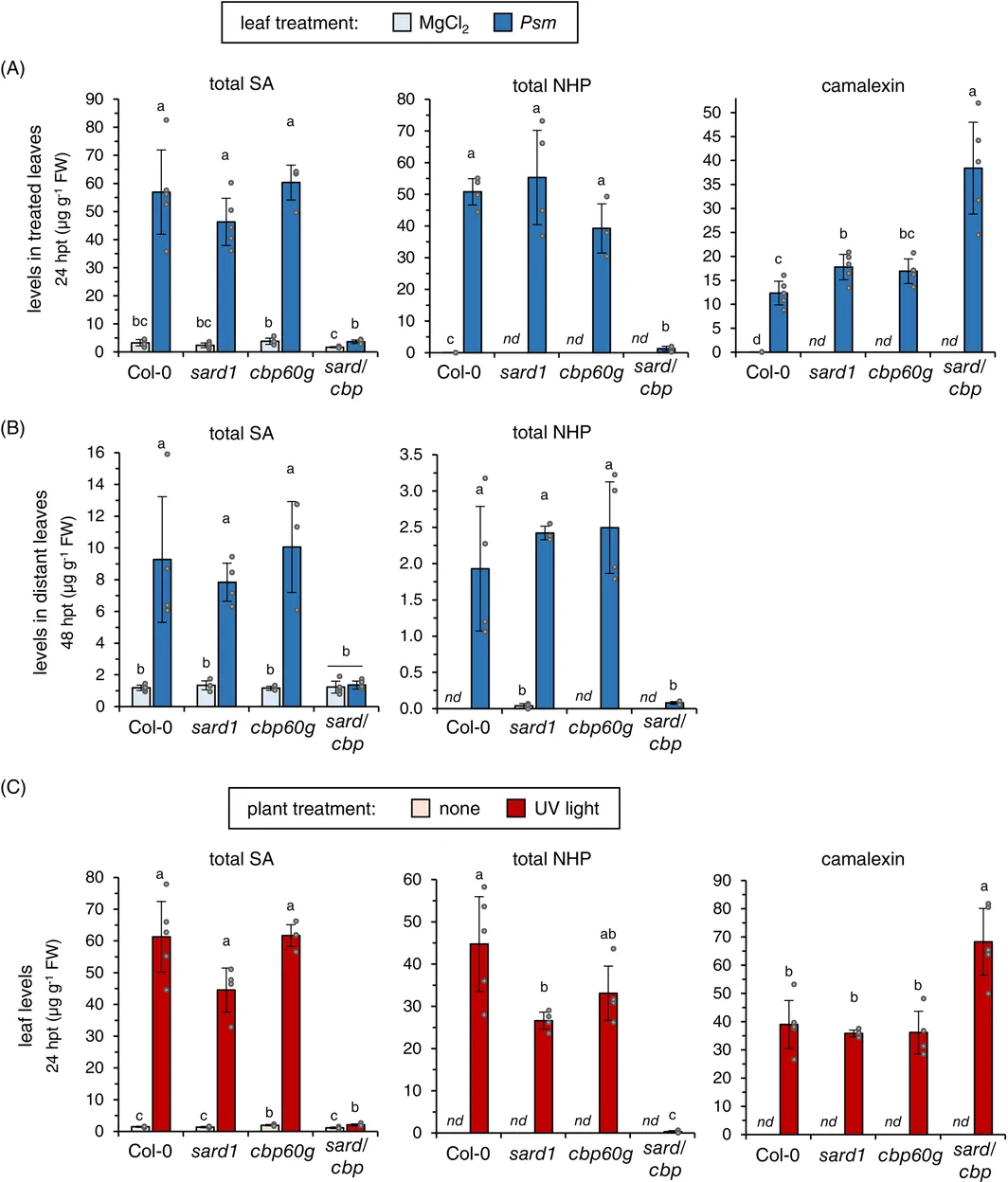
While the *sard/cbp60g* double mutant is defective in the *P. syringae*‐induced systemic accumulation of salicylic acid (SA) and N‐hydroxypipecolic acid (NHP) and their UV‐B light‐triggered biosynthesis, camalexin over‐accumulates in these plants under stress conditions. (A) Levels of total SA, total NHP, and camalexin in the *Psm*‐ or mock‐ (10 mM MgCl_2_) inoculated leaves of Col‐0 wild type, *sard1*, *cpb60g*, and *sard/cbp* plants at 24 h post‐treatment (hpt). Three leaves per plant were treated and combined for one biological replicate. Bars represent the means ± SD of three to five biological replicates. Metabolite levels are given in μg g^−1^ FW. Individual data points are super‐imposed on the bar graphs (gray circles). Different letters denote significant differences (*P* < 0.05, Kruskal–Wallis H‐test). See also Figure S3. (B) Levels of total SA and total NHP in distant (upper) leaves of Col‐0, *sard1*, *cpb60g*, and *sard/cbp* plants infiltrated in their lower leaves with a suspension of *Psm* or with mock‐solution. Three lower leaves of a given plant were *Psm*‐ or mock‐treated, and three upper leaves were harvested at 48 hpt and combined for one biological replicate. Bars represent the means ± SD of three to four biological replicates. Individual data points are super‐imposed on the bar graphs (gray circles). Different letters denote significant differences (*P* < 0.05, Kruskal–Wallis H‐test). Please note that camalexin does not accumulate in the systemic leaves of locally inoculated plants under these inoculation conditions. See also Figure S4. (C) Levels of total SA, total NHP, and camalexin in the leaves of the lines under investigation after exposing the plants to a UV‐B light stress period of 30 min. Control plants were left untreated. Four leaves per plant were harvested 24 h after the treatment and combined for one biological replicate. Bars represent the means ± SD of four to five biological replicates. Individual data points are super‐imposed on the bar graphs (gray circles). Different letters denote significant differences (*P* < 0.05, Kruskal–Wallis H‐test). See also Figure S5. Similar tendencies were observed in two independent experiments.

**Figure 6:**
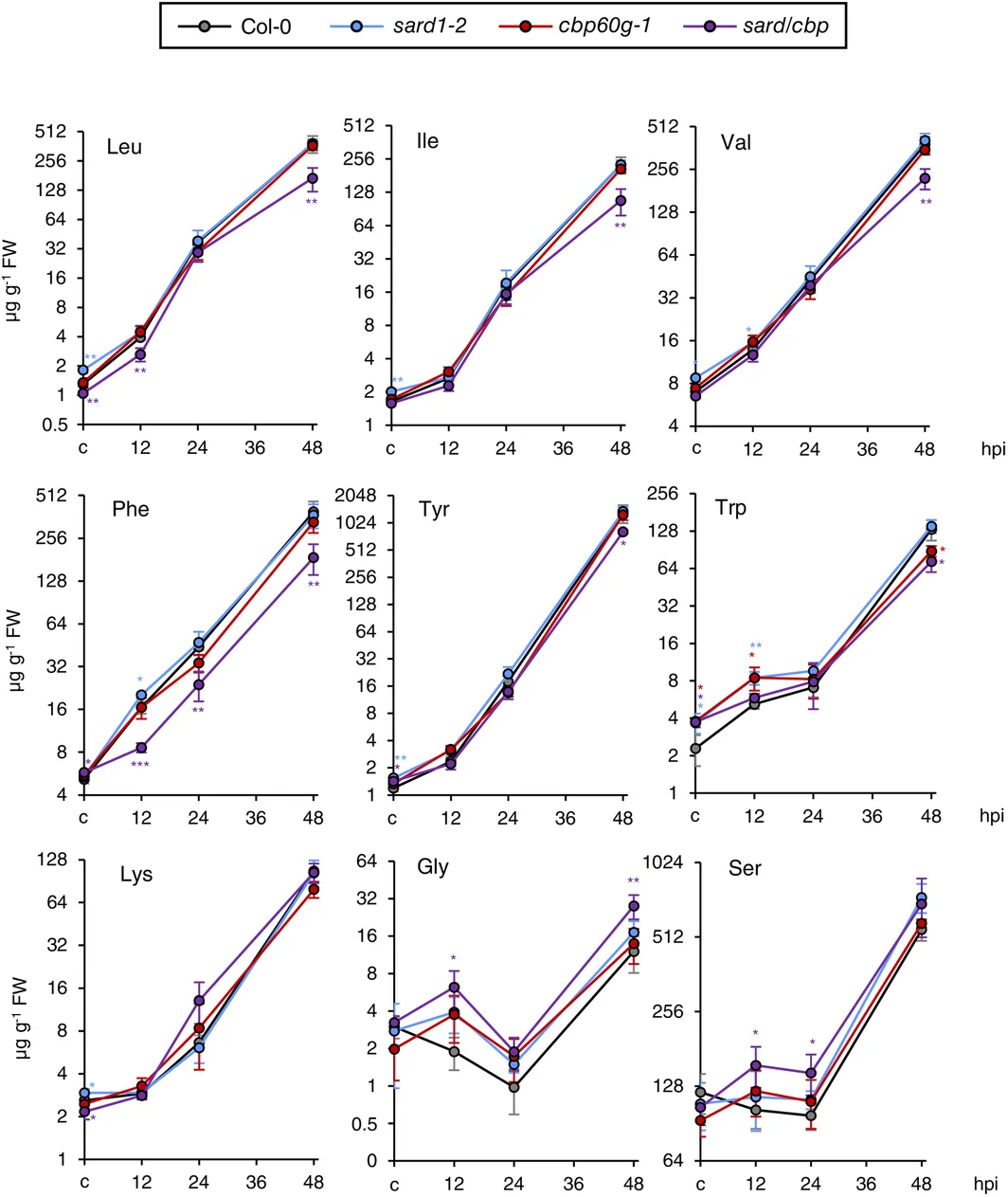
*P. syringae*‐induced accumulation of branched‐chain and aromatic amino acids is positively influenced by SARD1 and CBP60g. Time course analysis of the levels of different amino acid in *Psm*‐inoculated leaves of Col‐0 (gray), *sard1* (blue), *cpb60g* (red) and *sard/cbp* (purple) at 12, 24, and 48 hours post‐inoculation (hpi) compared with the levels in non‐inoculated, mock‐infiltrated leaves harvested at 12 h post‐treatment (c). Circles indicate the means (± SD) of three to five biological replicates, and asterisks next to the circles denote significant differences between mutant and Col‐0 values (***: *P* < 0.001, **: *P* < 0.01, *: *P* < 0.05; two‐tailed *t*‐test). Amino acid levels were determined by GC–MS analysis and given in μg g^−1^ FW. For other details, please refer to the legend of Figure 4. Two independent experiments were conducted that yielded similar tendencies.

Previous studies indicate that SARD1 and CBP60g positively regulate the expression of SA‐ and NHP biosynthetic genes in Arabidopsis upon bacterial inoculation, suggesting that SARD1/CBP60g also act upstream of NHP‐ and SA signaling in the SAR pathway. Consistently, it was shown that SA and the NHP precursor Pip accumulate to reduced levels in *sard/cbp* double mutants after pathogen attack (Sun et al., 2015, 2018; Wang et al., 2011; Zhang et al., 2010), although direct evidence of a requirement of functional SARD1/CBP60g for NHP accumulation and metabolism at the metabolic level was still missing. Since our transcriptome analyses indicated a diverse role of SARD1/CBP60g in defense‐related metabolism (Figure 3), we comparatively studied the metabolic changes that occur in Col‐0, *sard1*, *cbp60g*, and *sard/cbp* plants upon bacterial inoculation, ultraviolet (UV) light stress or NHP treatment in greater detail (Figures 4, 5, 6; Figures S3 to S5).

We first performed a time course analysis of *Psm*‐induced metabolite accumulation compared with mock‐control plants by collecting leaf samples at 12, 24, and 48 hours post‐inoculation (hpi) in inoculated leaves and at 48 hpi in distant, non‐treated leaves (Figures 4 and 5A,B and Figure S3). The levels of SA, the SA‐β‐glucoside (SAG), and the SA glucose ester (SGE) accumulated to high levels after bacterial attack in inoculated Col‐0 leaves over the period of investigation, with SAG representing the quantitatively major SA metabolite at later infection stages (48 hpi; Figure 4; Scholten et al., 2024). Although accumulation of SA and its two glucose conjugates was also high in the inoculated leaves of both single mutants, *cbp60g* showed a modestly reduced accumulation of the three quantified SA metabolites between 12 and 24 hpi, and *sard1* exhibited moderately reduced accumulation of both SAG and SGE at 24 hpi, respectively. By comparison, *sard/cbp* exhibited a much stronger deficit in the local biosynthesis of SA, since the double mutant only marginally accumulated SA, SAG, and SGE in the *Psm*‐inoculated leaves during the course of investigation (Figures 4 and 5A and Figure S3). Moreover, in contrast to the wild type and both of the single mutants, the *sard/cbp* double mutant was unable to increase the levels of SA and its glucose conjugates in distant, upper leaves of plants inoculated in lower leaves with *Psm* at 48 hpi (Figure 5B and Figure S4). Since the SA‐biosynthetic pathway can also be activated after exposure of Arabidopsis plants to UV light, we wondered whether pathway induction by UV light treatment would proceed via similar or different regulatory principles than induction by biotic stress. Twenty‐four hours after exposing entire plants for 30 min to UV‐B light, the leaves of Col‐0 accumulated substantial levels of SA, SAG, and SGE, and the *sard1* and *cbp60g* single mutants behaved similarly. In stark contrast, *sard/cbp* failed to increase free SA levels and only showed very mild increases of SAG and SGE following UV light exposure (Figure 5C and Figure S5), indicating that the deficiency of *sard/cbp* in accumulating SA and its derivatives is not specific to responses elicited by biotic stress stimuli.

The NHP precursor Pip accumulates to abundant levels in Arabidopsis leaves following *Psm* inoculation (Hartmann et al., 2018; Návarová et al., 2012). In our current time course analysis, the levels of Pip increased similarly in Col‐0, *sard1*, and *cbp60g* plants to about 100 μg g^−1^ fresh weight (FW) at 48 hpi (Figure 4B). Comparatively, Pip accumulated to reduced but substantial levels of about 40 μg g^−1^ FW in *Psm*‐inoculated leaves of *sard/cbp* at 48 hpi, indicating a partial dependence of inducible Pip biosynthesis on SARD1/CBP60g (Figure 4B; Sun et al., 2018). Moreover, NHP and the two glucose conjugates NHPG and NHPGE were substantially synthesized in *Psm*‐inoculated leaves of Col‐0, *sard1*, and *cbp60g* during the period of investigation, with *cbp60g* showing a slightly reduced accumulation of both NHP conjugates at 48 hpi (Figure 4B). The wild type and the *sard1* and *cbp60g* single mutant plants also significantly accumulated NHP and its conjugates in distant leaves of locally leaf‐inoculated plants (Figure 5B and Figure S4), as well as in the leaves of UV light‐exposed plants (Figure 5C and Figure S5). As observed for the SA metabolic pathway, *sard/cbp* showed by far the highest deficiencies regarding NHP biosynthesis and metabolism under all the examined conditions. First, the *Psm*‐induced accumulation of NHP and its glucose conjugates was reduced to very low levels at inoculation sites and fully suppressed in the systemic leaf tissue upon local bacterial attack in the *sard/cbp* double mutant (Figures 4 and 5A–C, Figures S3–S5). And second, the UV light‐induced generation of NHP and both of its conjugated forms was only marginally observed in *sard/cbp* plants (Figure 5C and Figure S5). Taken together, these analyses show that, irrespective of the applied stress stimulus, either functional *SARD1* or *CBP60g* is required for a substantial accumulation of SA and NHP, as well as the respective glucose‐conjugated forms, in Arabidopsis leaves.

The NHP‐induced expression of key biosynthetic genes of Trp‐derived secondary metabolism behaved differently than the induction of NHP and SA‐pathway genes in *sard/cbp* plants (Figure 3 and Figure S2). We therefore also assessed the stress‐induced accumulation of indolic compounds amenable to our GC–MS‐ and LC–MS‐based analyses. Camalexin, a major indolic phytoalexin in crucifers that is inducible under several biotic and abiotic stress conditions (Glawischnig, 2007; Müller et al., 2019), accumulated to substantial levels in both *Psm*‐inoculated Col‐0 leaves and the leaves of UV light‐exposed wild‐type plants (Figures 4A and 5A,B). Moreover, both *sard1* and *cbp60g* accumulated at least wild‐type‐like levels of camalexin upon biotic and abiotic stress treatments, with modest over‐accumulation in *Psm*‐inoculated *sard1* leaves. Remarkably, the leaves of the *sard/cbp* double mutant significantly over‐accumulated camalexin both after *Psm* inoculation and in response to UV light treatment (Figures 4A and 5A,C). In addition, the levels of three other detectable, pathogen‐inducible indolics, ICA, indole‐3‐carbaldehyde (ICC) and indole‐3‐carbonitrile (I3N) (Gamir et al., 2012; Pedras & Abdoli, 2013; Stahl et al., 2016), were analyzed in *Psm*‐inoculated and control leaves (Figure 4B). While ICA significantly accumulated to wild‐type‐like levels in the leaves of *sard1*, *cbp60g*, and *sard/cbp* plants, ICC over‐accumulated in both of the single and in the double mutant. Moreover, I3C showed a similar accumulation pattern in Col‐0, *sard1*, and *cbp60g* plants, and also over‐accumulated above wild‐type levels in the *sard/cbp* double mutant in the course of *Psm* infection (Figure 4B). Thus, as our gene expression data suggested (Figure 3 and Figure S2), the activation of indolic metabolism upon bacterial inoculation does not require functional SARD1/CPB60g, and even tends to be negatively regulated by these TFs. Therefore, in addition to our results on NHP‐inducible gene expression, the opposed mode of operation of SARD1/CPB60g in the regulation of NHP and SA metabolism on the one hand and Trp‐derived metabolism, on the other hand, also becomes apparent at the metabolic level in response to leaf inoculation by bacterial pathogens.

### SARD1 and CBP60g positively affect the accumulation of branched chain and aromatic amino acids as well as γ‐tocopherol following bacterial attack

We also examined a possible regulatory function of SARD1 and CBP60g in the *Psm*‐induced accumulation of canonical amino acids (Figure 6). As reported previously for Col‐0 (Návarová et al., 2012), the BCAAs Leu, Val, and Ile, the AAAs Phe, Tyr, and Trp, and the Pip and NHP precursor amino acid Lys accumulated significantly in response to *Psm* inoculation in the four Arabidopsis lines under investigation, and particularly high substance levels were detected at 48 hpi. More specifically, while Col‐0, *sard1*, and *cbp60g* accumulated BCAAs, AAAs, and Lys to overall similar levels, the accumulation of Leu, Ile, Phe, and Trp at 48 hpi was about twofold lower for *sard/cbp* than for Col‐0. Moreover, Val and Tyr accumulation was reduced by approximately 1.5‐fold in the double mutant compared with the wild type (Figure 6). By contrast, Lys accumulated to similar levels in *sard/cbp* and the other examined Arabidopsis lines, and the levels of Ser and Gly were even higher for some time points in the leaves of the *sard/cbp* double mutant than in wild‐type leaves (Figure 6). Therefore, SARD1 and CBP60g specifically exert a positive influence on the accumulation of BCAAs and AAAs, and thereby again show a redundant mode of action. Quantitatively, however, the dependency of BCAA and AAA accumulation on SARD1/CBP60g was not as pronounced as for the activation of the SA and NHP‐metabolic pathways.

Two other substances strongly biosynthesized in the leaves of Arabidopsis plants in response to *P. syringae* inoculation are the Tyr‐derived antioxidant γ‐tocopherol and the unsaturated sterol stigmasterol (Griebel & Zeier, 2010; Stahl et al., 2019). While γ‐tocopherol accumulated in both Col‐0 and *sard1* plants to approximately 16 μg g^−1^ FW at 48 h after *Psm* inoculation, its accumulation was reduced by about fourfold in the *sard/cbp* double and about twofold in the *cbp60g* single mutant (Figure 4B). Stigmasterol is generated by desaturation of β‐sitosterol by the cytochrome P450 enzyme CYP710A1, and the ratio of the contents of stigmasterol to β‐sitosterol is a good indicator of β‐sitosterol formation in Arabidopsis leaves (Griebel & Zeier, 2010). The observed ratio of the levels of stigmasterol and β‐sitosterol increased similarly in Col‐0, *sard1*, *cbp60g*, and *sard/cbp* plants (Figure 4B). Thus, *SARD1/CBP60g* amplifies the biosynthesis of γ‐tocopherol but not the generation of stigmasterol in *P. syringae*‐inoculated Arabidopsis leaves.

### SARD1 and CBP60g boost the induced expression of NHP‐ and SA‐biosynthetic genes in pathogen‐inoculated, local leaves and are indispensable for their increased expression in distant, systemic leaves

**Figure 7:**
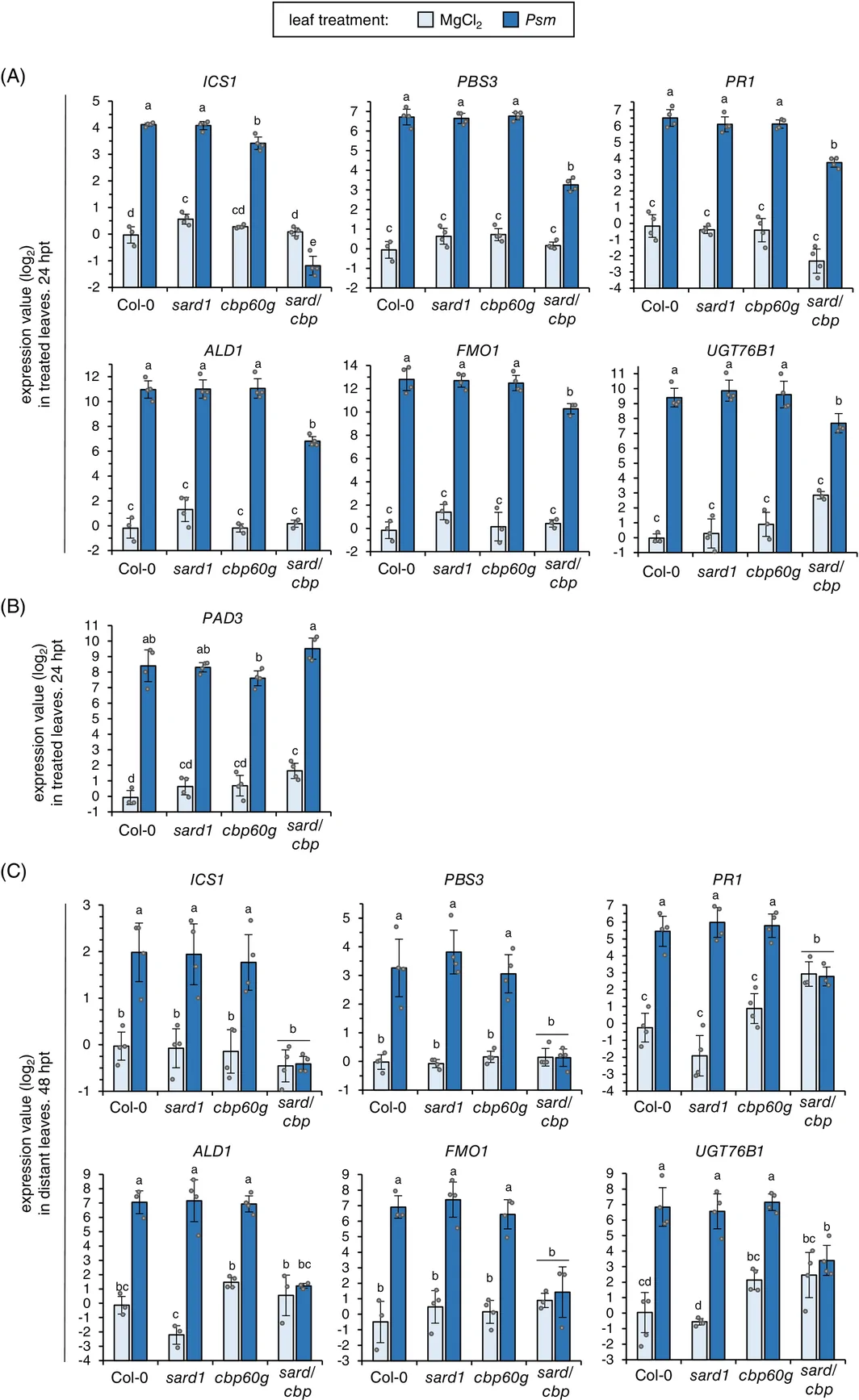
SARD1 and CBP60g promote the *P. syringae*‐induced expression of salicylic acid (SA) and N‐hydroxypipecolic acid (NHP)‐pathway genes in locally inoculated leaves and enable transcript elevation in systemic leaf tissue, but are dispensable for pathogen‐induced expression of the camalexin biosynthetic gene *PAD3*. (A) Transcript levels of *ICS1, PBS3, PR1, ALD1, FMO1*, and *UGT76B1* in the inoculated leaves of Arabidopsis Col‐0 (wild type), *sard1*, *cpb60g*, and *sard/cbp* at 24 hours post‐inoculation (hpi) compared with the levels in mock‐treated leaves. (B) Transcript levels of *PAD3* at 24 hpi in inoculated leaves. (C) Transcript levels of *ICS1, PBS3, PR1, ALD1, FMO1*, and *UGT76B1* in distant, upper leaves 48 h of Arabidopsis plants treated in their lower leaves with *Psm* or mock‐solution. Please refer to Figure 5 for experimental details. Expression values were determined by RT‐qPCR relative to *POLYPYRIMIDINE TRACT*‐*BINDING PROTEIN 1* (*PTB1*) as a reference gene (Czechowski et al., 2005) and expressed relative to the mean values of the Col‐0 mock‐sample. Bars represent the mean of log_2_‐transformed values ± SD of three to four biological replicates. Individual data points are superimposed on the bar graphs (gray circles). Different letters denote significant differences (*P* < 0.05, Kruskal–Wallis H‐test). Two independent experiments yielding similar tendencies were conducted.

To complete our analyses on the opposed mode of action of SARD1/CPB60g in SAR‐associated gene expression and metabolism, we examined the *Psm*‐induced expression of the key SA‐ and/or NHP‐pathway genes *ICS1, PBS3, ALD1, FMO1, UGT76B1*, and *PR1* at sites of bacterial attack and in systemic leaf tissues by reverse transcriptase‐polymerase chain reaction (RT‐qPCR) analysis. Collectively, upon *Psm* inoculation of Col‐0 plants, expression of all of the mentioned genes was strongly upregulated in locally attacked leaves at 24 hpi and also significantly enhanced in distant, systemic leaves at 48 hpi. The *sard1* and *cbp60g* single mutants again behaved similarly than the wild type in inducing both local and systemic gene expression (Figure 7). By contrast, validating previous results on *ICS1* and *PR*‐*1* expression (Wang et al., 2011; Zhang et al., 2010), the pathogen‐induced expression of the SA‐ and NHP‐pathway‐associated genes was significantly attenuated in the *sard/cbp* double mutant in the locally attacked tissue (Figure 7A). Remarkably, the *Psm*‐induced systemic elevation of the transcript levels of these SAR‐relevant genes was fully suppressed in the *sard/cbp* double mutant (Figure 7C), underscoring the important function of SARD1/CPB60g in the overall SAR response (Figure 1A,B). However, unlike expression of the SA‐ and NHP‐pathway genes, the *Psm*‐triggered induction of the key camalexin biosynthetic gene *PAD3* was at least as strong in the *sard/cbp* mutant than in the wild type (Figure 7B). Thus, the opposed influence of SARD1/CPB60g on NHP/SA‐related and indolic defense pathways in Arabidopsis is also apparent at the level of *P. syringae*‐induced gene expression.

### SARD1/CBP60g oppositely affect the NHP‐mediated priming of pathogen‐induced salicylic acid and camalexin biosynthesis

The SAR state enables plants to more vigorously defend themselves against subsequent pathogen attack, a phenomenon often designated as immune priming. Upon biological induction of SAR, endogenously accumulating NHP is the decisive trigger that establishes an immune‐primed state (Zeier, 2021). Similar to the biologically induced SAR state, exogenous treatment with NHP is sufficient to prime plants for enhanced pathogen‐ or molecular pattern‐induced responses, which characteristically include SA biosynthesis and camalexin accumulation (Löwe et al., 2023; Yildiz et al., 2021; Zeier, 2021).

**Figure 8:**
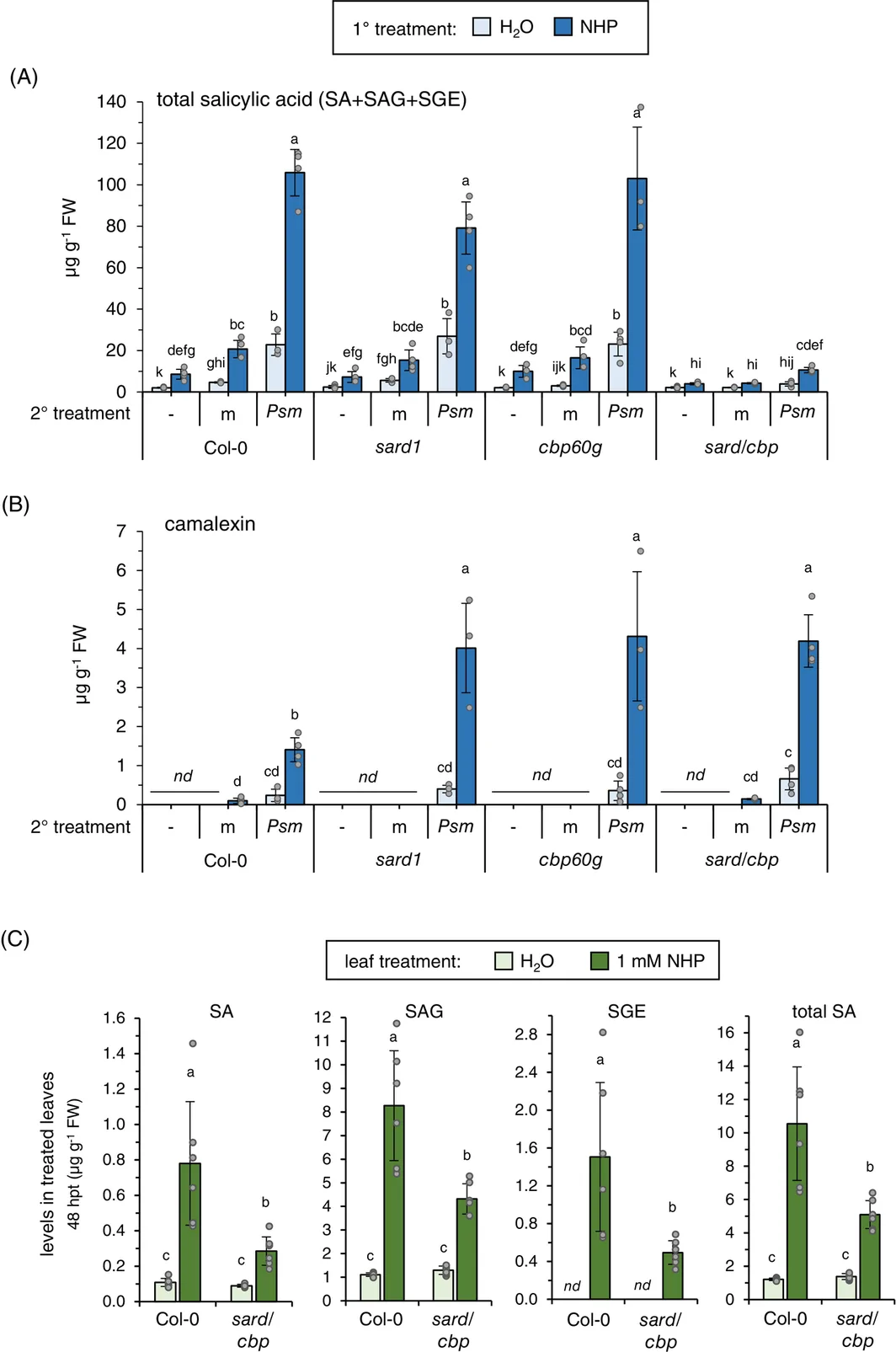
SARD1 and CPB60g oppositely affect the N‐hydroxypipecolic acid (NHP)‐induced priming of pathogen‐induced salicylic acid (SA) and camalexin accumulation. (A, B) NHP‐induced priming of the pathogen‐induced accumulation of total SA (defined as the sum of SA, SA‐β‐glucoside and SA glucose ester) (A) and of camalexin (B) in Col‐0, *sard1*, *cpb60g*, and *sard/cbp* plants. As a 1° treatment, plants were watered with 10 ml of 1 mM NHP (induction of priming) or 10 ml of H_2_O (control). One day later, three leaves of a given plant were infiltrated with a suspension (OD 0.005) of *Psm* bacteria (*Psm*) or mock‐treated by infiltrating 10 mM MgCl_2_ solution (m). The metabolite levels of leaves were determined 12 h after the 2° treatment. The leaves of a third set of plants were left untreated with respect to the 2° treatment (−), and leaf samples were harvested at the same time as those of the 2°‐treated plants. One replicate sample consisted of three leaves from one plant. Bars represent means ± SD of three or four biological replicates. Metabolite levels are given in μg g^−1^ FW. Individual data points are superimposed on the bar graphs (gray circles). Different letters denote significant differences (*P* < 0.05, Kruskal–Wallis H‐test). (C) Levels of SA, SAG, SGE, and total SA in the leaves of plants infiltrated with 1 mM NHP or water 48 h post‐treatment. Three leaves of a given plant were treated and combined for one biological replicate, and six replicates were analyzed.

To investigate the role of SARD1 and CBP60g in the NHP‐induced priming response, we employed a previously established priming assay on Col‐0, *sard1*, *cbp60g*, and *sard/cbp* plants (Figure 8; Yildiz et al., 2021). We therefore watered individual plants with 10 ml of 1 mM NHP or H_2_O as inducing or control pre‐treatments (1° treatments), respectively, challenge‐infected plants with *Psm* as a 2° treatment 1 day later, and collected the 2°‐challenged leaves 12 h after the treatment for metabolite analysis. Leaves of 2° mock‐treated plants and leaves of plants that lacked the 2° treatment served as further controls. As observed previously (Yildiz et al., 2021), the NHP‐pre‐treatment alone was sufficient to elevate total SA levels to some extent, and also modestly primed total SA accumulation upon the 2°‐mock treatment in Col‐0 plants. Significantly, while *Psm* challenge of H_2_O‐pre‐treated plants triggered only moderate elevations of total SA levels at 12 hpi, NHP‐pre‐treatment strongly boosted the *Psm*‐triggered accumulation of total SA (Figure 8). This NHP‐induced priming for *Psm*‐induced SA biosynthesis was similar in Col‐0, *sard1* and *cbp60g* plants. Notably, the NHP‐induced priming of *Psm*‐ (and mock‐treatment‐) induced SA biosynthesis was almost absent in *sard/cbp* double mutants (Figure 8A). In addition, the NHP‐induced direct accumulation of SA (and of the glucose conjugates SAG and SGE) was attenuated in the *sard/cbp* plants, irrespective of whether application of the NHP solution was performed via soil watering or leaf infiltration (Figure 8A,C). These observations are in agreement with the weakened NHP‐induced expression of SA biosynthesis genes in the double mutant (Figure 3 and Figure S2A).

An Arabidopsis response characteristically primed by NHP is the pathogen‐ or elicitor‐induced accumulation of camalexin (Löwe et al., 2023; Yildiz et al., 2021). Consistent with previous results, we observed a significant priming of the *Psm*‐induced accumulation of camalexin in the Col‐0 wild type, but no direct induction of camalexin accumulation by NHP treatment alone. Remarkably, compared to Col‐0, the NHP‐mediated priming of *Psm*‐induced camalexin accumulation was enhanced in *sard1*, *cbp60g* and *sard/cbp* plants (Figure 8B). Therefore, SARD1/CPB60g also oppositely affected the NHP‐induced priming of pathogen‐triggered SA and camalexin biosynthesis, respectively.

## DISCUSSION

### The SARD1/CBP60g transcriptional node is crucial for the metabolism of the SAR‐inducing defense hormones NHP and SA but does not support indolic pathway activation

**Figure 9:**
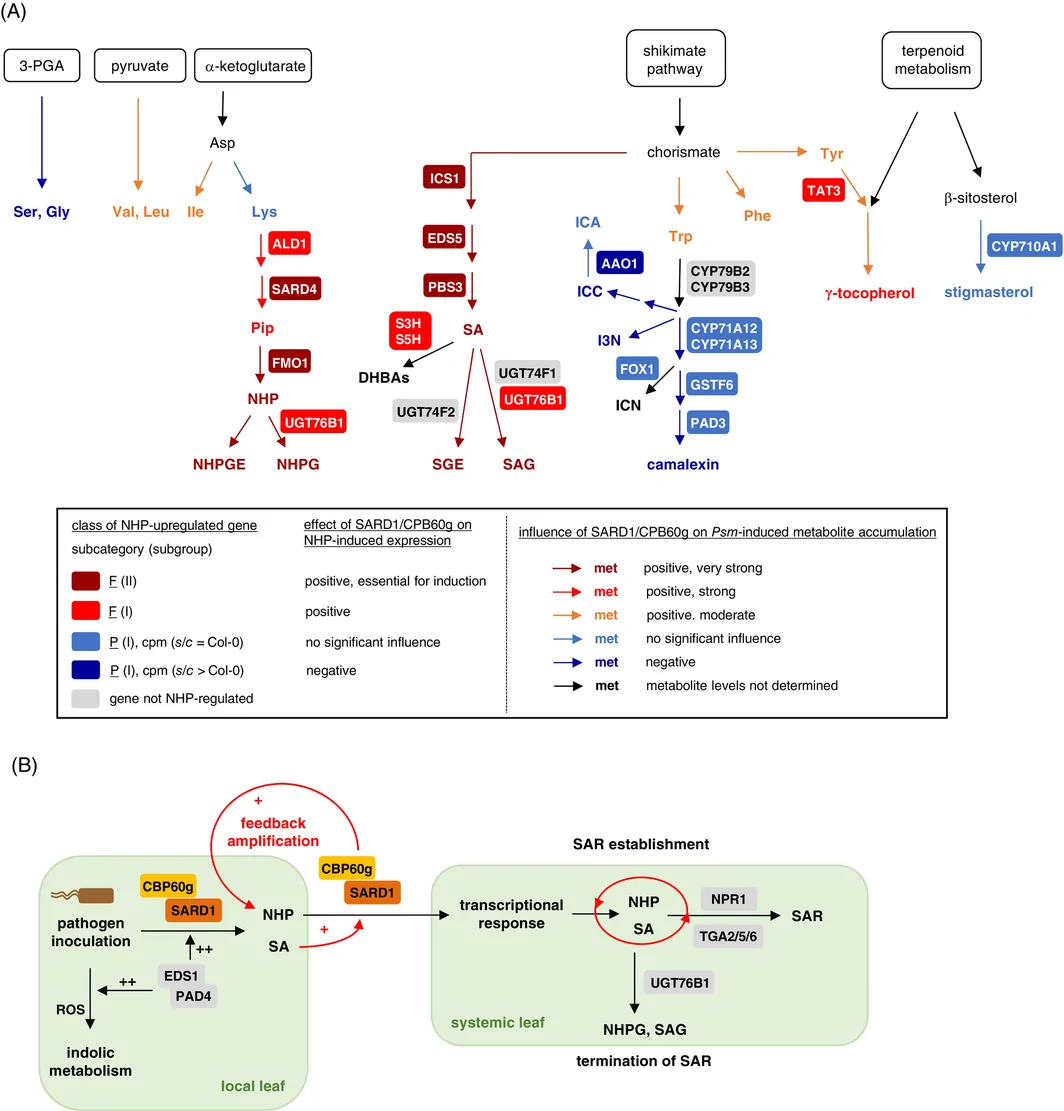
Transcription factors SARD1 and CPB60g act redundantly and selectively in the regulation of N‐hydroxypipecolic acid (NHP) and salicylic acid (SA) metabolism, as well as downstream of NHP to guarantee systemic acquired resistance. (A) Overview of key metabolic pathways activated in pathogen‐inoculated Arabidopsis leaves and the regulatory influence of SARD1/CPB60g on these pathways, both at the level of NHP‐inducible gene expression and metabolite accumulation. Genes whose NHP‐induced expression and metabolites whose *P. syringae*‐induced accumulation show different dependency on SARD1/CPB60g are depicted by different colors. See legend for details. The degree of influence of SARD1/CPB60g on the levels of accumulating metabolites is defined as very strong (ratios Col‐0‐to‐*sard/cbp* at 24 and/or 48 hpi >6), strong (ratio between 6 and 3), and moderate (ratio between 3 and 1.5); see also Figures 4 and 6. (B) The SARD1/CPB60g module functions in the transcriptional regulation of NHP and SA biosynthesis, the promotion of NHP‐mediated systemic acquired resistance (SAR) establishment, and the termination of SAR. See discussion section for further details.

Pathogen‐inducible metabolic pathways in Arabidopsis leaves are diverse and include accumulation of aromatic and branched‐chain amino acids, activation of the biosynthesis and catabolism of the immune‐regulatory metabolites NHP and SA, induction of Trp‐derived metabolism toward production of indolic defense compounds, accumulation of Tyr‐derived γ‐tocopherol, and generation of the unsaturated phytosterol stigmasterol (Figure 9A). In the current study, we have investigated the roles of the TFs SARD1 and CPB60g in the metabolic reprogramming of Arabidopsis leaves inoculated with the compatible bacterial pathogen *Pseudomonas syringae* pv. *maculicola* by comparing wild‐type immune responses to those of the previously identified single and double knockout mutants *sard1*, *cbp60g*, and *sard/cbp* (Wang et al., 2011; Zhang et al., 2010). SARD1 and CBP60g represent two closely sequence‐related, plant‐specific TFs that are highly induced upon pathogen inoculation or exogenous treatment with pathogen‐associated molecular patterns, such as flagellin. While CBP60g requires calmodulin‐binding activity for its function, SARD1 does not function as a calmodulin‐binding protein (Wang et al., 2009, 2011; Zhang et al., 2010).

When comparing the general accumulation patterns of diverse metabolites in the leaves of *P. syringae*‐inoculated plant lines under investigation, we detected high similarities between Col‐0 wild type and *sard1* or *cbp60g* single mutant plants, while *sard/cbp* double mutants showed strong specific alterations in these pathogen‐triggered metabolic shifts (Figures 4, 5, 6, Figure S3). This indicates that SARD1 and CBP60g act primarily in a redundant manner in influencing the pathogen‐inducible metabolic reprogramming in inoculated Arabidopsis leaves, and that one of the TFs can compensate for the other in the respective single mutants. This functional redundancy of the two TFs was previously reported in context with the function of SARD1 and CBP60g in the regulation of pathogen‐ or flagellin‐induced SA biosynthesis (Wang et al., 2011; Zhang et al., 2010), and our data indicate that SARD1 and CBP60g also redundantly affect UV light stress‐induced metabolic changes (Figure S5).

Our comprehensive metabolic analyses of the *sard/cbp* double mutant reveal that SARD1/CBP60g exert distinct influences on specific pathogen‐inducible metabolic pathways in the inoculated Arabidopsis leaf (Figure 9A). A first striking feature is that the *Psm*‐induced biosynthesis and metabolism of SA strongly depend on functional SARD1/CBP60g, as illustrated by the markedly compromised accumulation of free SA and its two main derivates, the glucoside SAG and the glucose ester SGE in the *P. syringae*‐inoculated leaves of the double mutant. For example, accumulation of total SA as the sum of the three above‐mentioned SA forms only amounted to about 4% of the wild‐type levels in the *sard/cbp* double mutant at 48 hpi, while the two single mutants only showed moderately reduced induction of *Psm*‐triggered SA biosynthesis (Figures 4 and 5, Figure S3). Previous studies report on this role for SARD1/CBP60g in SA biosynthesis by assessing the levels of free and bound SA forms in Arabidopsis leaves, but without further specifying the nature of SA conjugates (Wang et al., 2009, 2011; Zhang et al., 2010). We also confirmed that the compromised accumulation of SA and its two main glucose conjugates goes hand in hand with a significant reduction in *Psm*‐induced increases in transcript levels of SA‐biosynthetic genes *ICS1* and *PBS3*, the SA glycosyltransferase gene *UGT76B1*, as well as *PR1*, which is generally considered a marker gene for an activated SA‐signaling pathway (Figure 7; Sun et al., 2015; Wang et al., 2009, 2011; Zhang et al., 2010). Thus, it is reasonable to assume that the strong dependency of SA‐related metabolism on functional SARD1/CBP60g is directly related to the function of these TFs in gene transcription. Electrophoretic mobility shift assays, chromatin immunoprecipitation (ChIP)‐PCR, and/or ChIP sequencing analysis indicate that both TFs bind to the promoters of the SA biosynthesis genes *ICS1*, *EDS5*, and *PBS3*, corroborating this assumption (Zhang et al., 2010; Wang et al., 2011; Sun et al., 2015).

Another conclusive result of our metabolite analyses is that the SARD1/CBP60g transcriptional node possesses a central function in the biosynthesis and metabolism of the critical SAR‐regulator NHP (Figure 9A). We directly show that the accumulation of NHP in *P. syringae*‐inoculated leaves is heavily attenuated in the *sard/cbp* double mutant (Figures 4 and 5, Figure S3). Similarly, the strong accumulation of the NHP glucoside NHPG and the glucose ester NHPGE observed in the wild type are greatly reduced in *sard/cbp* (Figures 4 and 5, Figure S3). Quantitatively, the sum of the three assessed NHP forms accumulated to merely 3% of the sum of the corresponding wild‐type levels at 48 h after *P. syringae* inoculation, indicating that the positive regulatory effect of SARD1/CBP60g on the biosynthesis and glucose conjugation of NHP is similarly stringent to its influence on SA‐related metabolism. In terms of NHP metabolism, the single mutants were again hardly affected, since *cbp60g* only showed a small and *sard1* no reduction of total NHP accumulation at 48 hpi (Figure 4A). Although *sard/cbp* also exhibited a significantly reduced biosynthesis of the NHP precursor Pip, Pip accumulation still occurred substantially in the double mutant (about 37% of wild‐type levels) and thus depended less stringently on the SARD1/CBP60g regulatory node than NHP accumulation. Consistently, a previous study assessed reduced accumulation of Pip in *P. syringae*‐inoculated leaves of the *sard/cbp* double mutant at early infection stages (Sun et al., 2018), and transcript accumulation of the Pip (and NHP) biosynthetic gene *ALD1* was only partially suppressed in inoculated *sard/cbp* leaves (Figure 7A). Moreover, the strongly compromised generation of NHP and NHP glucose conjugates in *sard/cbp* was also paralleled by a significantly reduced *P. syringae*‐induced elevation of transcript levels of the NHP biosynthetic gene *FMO1* and the NHP glucosyltransferase gene *UGT76B1* (Figure 7A). In addition, it was shown by ChIP‐PCR analysis that SARD1 and CBP60g directly target the promoters of both *ALD1* and *FMO1* (Sun et al., 2015). Together, these data demonstrate that SARD1/CBP60g exert strong positive effects on NHP biosynthesis and metabolism, which appears to be primarily executed at the level of gene transcription. Because of the marked residual *P. syringae*‐induced increase of transcript levels of the NHP‐metabolic genes (Figure 7A), however, the impairment in regulatory or post‐transcriptional events might also contribute to the observed NHP deficiency of *sard/cbp*. It is interesting to note in this context that Lys, the metabolic precursor of Pip and NHP, accumulates to wild‐type‐like levels in *sard/cbp* (Figure 6), suggesting that the regulatory influence of the SARD1/CBP60g transcriptional node is not related to the metabolic events that cause the biotic stress‐inducible biosynthesis of Lys but starts at the stage of Lys catabolism (Figure 9A).

According to our analytical results, another major pathogen‐inducible metabolic route in Arabidopsis leaves, the catabolism of Trp that culminates in the generation of different indolic metabolites (Müller et al., 2015; Zhao et al., 2002), can very effectively proceed without functional SARD1/CBP60g (Figure 9A). A major sub‐pathway in this regard enables the accumulation of the phytoalexin camalexin (Glawischnig, 2007; Nafisi et al., 2007; Schuhegger et al., 2006), but other indolics such as ICN, ICA, ICC, and I3N have also been found to increase to substantial levels in *P. syringae*‐inoculated leaves (Gamir et al., 2012; Pedras & Abdoli, 2013; Rajniak et al., 2015; Stahl et al., 2016). Without exception, the indolic compounds detected with the here‐applied GC–MS and LC–MS‐based analytical procedures (camalexin, ICA, ICC, and I3N) accumulated to wild‐type‐like or to even higher levels in the *sard/cbp* double mutant (Figure 4). The same appeared true for the indolic cyanide ICN, which we detected by LC–MS‐based analysis as a prominently accumulating metabolite but nevertheless did not quantify, since we noticed, in accordance with previous work (Rajniak et al., 2015), that a relatively rapid degradation of this metabolite occurs in methanolic extracts. Remarkably, although the amino acid precursor Trp accumulated to lower levels at 48 hpi in the leaves of *sard/cbp* compared to wild‐type leaves, camalexin, ICC, and I3N even tended to over‐accumulate in the double mutant (Figures 4, 5A and 6, Figure S3), and a similar tendency existed for *P. syringae*‐induced accumulation of *PAD3* transcript levels (Figure 7B). These results suggest a measurable negative influence of SARD1/CBP60g at the level of Trp catabolism toward generation of indolic defense metabolites (Figure 9A). Notably, the opposed impact of SARD1/CBP60g on NHP and SA metabolism on the one hand and indolic metabolism on the other hand was observed for both the *P. syringae* and UV light‐induced response and is thus valid for plant reactions to both biotic and abiotic types of stress (Figure 5C).

### As a mediator of both the biosynthesis of and downstream responses to NHP and SA, the SARD1/CBP60g node emerges as a central element in signal amplification during SAR establishment

Previous studies have established that SARD1/CBP60g possess important roles in plant local resistance to compatible and incompatible leaf pathogens, induction of enhanced leaf resistance in response to the bacterial elicitor flagellin, and induction of SAR by different bacterial pathogens. Again, SARD1 and CBP60g act redundantly to mediate these forms of resistance, since the most significant immune defects are generally observed in *sard/cbp* double mutants compared to the respective single mutants (Sun et al., 2015; Wang et al., 2009, 2011; Zhang et al., 2010; Figure 1, Figure S1). However, while some of the previously conducted resistance assays reveal moderately attenuated defects in basal immunity to *Psm* infection also in *sard1* and *cbp60g* (Wang et al., 2011), our assays consistently indicate a wild‐type‐like basal immunity of both single mutants at the resistance level (Figure 1, Figure S1). These differences might be related to plant growth or pathogen inoculation conditions.

Consistent with the previous findings, SAR establishment following a localized inducing inoculation with *Psm* was severely compromised in the *sard/cbp* double mutant in our assays, while the single mutants were both able to trigger a wild‐type‐like SAR (Figure 1B). Since the pathogen‐triggered accumulation of both NHP and SA in plants is required for the establishment of an effective SAR response (Bernsdorff et al., 2016; Hartmann et al., 2018; Mishina & Zeier, 2006; Návarová et al., 2012; Nawrath & Métraux, 1999; Wildermuth et al., 2001), it is reasonable to assume that the compromised biosyntheses of NHP and SA in inoculated *sard/cbp* leaves largely contribute to its defects in acquired resistance (Figures 1, 4 and 5, Figure S3).

A main aim of this study was to clarify whether SARD1/CBP60g would also function downstream of NHP in the induction of SAR and the associated transcriptional response. Our results show that SAR is triggered to significantly lower amounts in distant leaves of locally NHP‐treated *sard/cbp* plants than in wild‐type plants, while the single mutants both showed a wild‐type‐like resistance response (Figure 1A). In addition, the NHP‐induced transcriptional SAR response proved to be markedly attenuated in the *sard/cbp* double mutant, while showing only modest reduction in the *sard1* single mutant and a wild‐type‐like magnitude in *cbp60g* (Figures 2 and 3; Data S1). Thus, primarily in a redundant manner, SARD1/CBP60g also contributes to the events leading to defense gene expression and resistance induction downstream of NHP generation in the SAR response. Similarly, SARD1/CBP60g also acted redundantly to translate elevated SA levels into acquired resistance (Figure 1C). The dual roles in both NHP/SA biosynthesis and downstream action predestines the SARD1/CBP60g node of exerting a major role in a previously proposed feedback amplification mechanism that is based on positive NHP‐ and SA‐interplay and supposedly contributes to conveying SAR signal transduction from the locally inoculated to distal, non‐inoculated leaves (Figure 9B; Zeier, 2021).

A synergistic interplay of NHP and SA can be observed at several levels (Zeier, 2021). For instance, early accumulation of NHP in the Arabidopsis‐*P. syringae* interaction as well as NHP‐induced downstream resistance responses are boosted by a functional SA‐biosynthetic pathway, which indicates amplification of NHP biosynthesis and signaling by SA (Figure 1A; Bernsdorff et al., 2016; Löwe et al., 2023; Yildiz et al., 2021). Conversely, NHP primes plants for enhanced SA biosynthesis and SA‐inducible *PR1* expression (Yildiz et al., 2021). Notably, we show here that the SARD1/CBP60g node is essential for the NHP‐induced priming of pathogen‐triggered SA biosynthesis and therefore mediates an important aspect of the positive interplay between NHP and SA in immune signal amplification (Figure 8A).

The particular importance of the SARD1/CBP60g module for SAR also manifests itself in our findings that all of the examined *P. syringae*‐induced systemic responses strictly depend on this transcriptional node: The pathogen‐induced elevation of transcripts of the SAR marker gene *PR1* and the key SA and NHP‐metabolic genes in leaves distant from inoculation sites, that were observed to similar high levels in wild type, *sard1*, and *cbp60g*, proved to be completely absent in the *sard/cbp* plants (Figure 7C). And this was accompanied by an almost complete failure of the double mutant to elevate SA and NHP levels in systemic tissue upon localized pathogen contact (Figure 5B and Figure S4). This virtual absence of systemic resistance responses in the double mutant might be the consequence of the markedly reduced accumulation of the SAR inducers NHP and SA in inoculated leaves and a therefore strongly diminished possibility of metabolic transport into distant leaves. Alternatively, it might result from a blockage of the above‐discussed NHP‐triggered and SARD1/CBP60g‐promoted feedback amplification loop that would otherwise result in enhanced systemic SAR gene expression and, consequently, in *de novo* biosynthesis of NHP and SA in the systemic leaf tissue (Figure 9B).

### Relation to other regulatory units of the NHP‐ and SA‐mediated SAR pathway

Previous studies have characterized several other regulatory elements of NHP biosynthesis and signal transduction. For example, the two interacting immune‐regulatory proteins EDS1 and PAD4 not only amplify the biosynthesis of SA (Feys et al., 2001; Zhou et al., 1998) but also boost the generation of NHP, NHPG, and NHPGE (Bauer et al., 2021; Hartmann et al., 2018). On the basis of detailed expression profiling analyses, the EDS1/PAD4 node has been placed upstream of the function of SARD1/CBP60g in the Arabidopsis basal immune response to *P. syringae* (Wang et al., 2011), which is consistent with the overlapping roles of the EDS1/PAD4 and SARD1/CBP60g hubs in regulating NHP and SA metabolism following pathogen perception (Figure 9B). Moreover, the class I TGA TFs TGA1 and TGA4 positively affect the accumulation of NHP and SA (derivates) in pathogen‐inoculated plants and directly modulate the expression of SARD1 and CBP60g (Sun et al., 2018; Yildiz et al., 2023). Furthermore, Ca^2+^‐related signal transduction appears to play an important role in the SARD1/CBP60g‐mediated biosynthesis of NHP and SA. On the one hand, Ca^2+^ stimulated the binding of calmodulin to CBP60g, and complementation analyses of *cbp60g* mutants with point‐mutated *CBP60g* versions indicated that calmodulin binding is necessary for strong pathogen‐induced SA accumulation (Wang et al., 2009). On the other hand, overexpression of the Ca^2+^‐dependent protein kinase gene *CPK5* in Arabidopsis resulted in a *SARD1*‐dependent enhancement of basal SA and NHP levels and an *FMO1*‐dependent elevation of *ICS1* transcript levels (Guerra et al., 2020), suggesting an involvement of CPK5 in the above‐discussed signal relay in SAR.

The SA‐receptor NPR1 and the Class II TGA TFs TGA2, TGA5, and TGA6 are other important components of the Arabidopsis SAR response (Cao et al., 1997; Ding et al., 2020; Zhang et al., 2003). Both NPR1 and the redundantly acting TGA factors primarily operate as key downstream components of the SAR‐activating metabolites SA and NHP (Yildiz et al., 2021, 2023). In contrast to the SARD1/CBP60g node, which is required for a part but not the full set of NHP‐triggered SAR gene expression (Figures 2 and 3), NPR1 and TGA2/5/6 are essential for virtually the entire NHP‐triggered transcriptional response and seem to primarily control SAR gene transcription systemically in the plant during the biologically induced SAR process. At the same time, SARD1/CBP60g possess additional importance for the inducible NHP and SA biosynthesis at pathogen inoculation sites (Figures 4 and 5, Figures S3 and S4), for which NPR1 and TGA2/5/6 are not required (Figure 9B; Yildiz et al., 2021; Yildiz et al., 2023).

### A double‐edged role of the SARD1/CBP60g node in the NHP‐induced transcriptional response

A main goal of the current study was to examine at the whole‐genome level whether the SARD1/CBP60g node would be involved in the transcriptional response toward elevated NHP (Figures 2 and 3, Figure S2). Our RNA‐seq‐based analysis shows that SARD1/CBP60g is required for a significant NHP‐triggered upregulation of about half (271 Subgroup II) of the 558 NHP‐inducible Col‐0^+^ genes (Figure 2A,C). A subsequent classification procedure further defined two subcategories among the Col‐0^+^ group containing genes, whose NHP‐induced expression either exhibits a very pronounced dependency on SARD1/CBP60g (subcategory F) or whose NHP‐induced upregulation does not require functional SARD1/CBP60g (subcategory P) (Figure 2D).

Notably, our analysis on NHP‐inducible gene expression revealed a virtually identical picture of the double‐edged role of SARD1/CBP60g in immune‐related metabolism compared with our analytical‐chemical assessment of *P. syringae*‐ or UV light‐inducible metabolites. On the one hand, the SARD1/CBP60g node boosts the NHP‐triggered expression of a specific subset of SAR genes that are central for the biosynthesis and metabolism of the SAR‐regulatory metabolites NHP (*ALD1*, *SARD4*, *FMO1*, and *UGT76B1*) and SA (*ICS1*, *EDS5*, *PBS3, UGT76B1*, *S3H*, and *S5H*), as well as expression of other SAR‐associated genes (e.g., *EDS1 and PR1*) (Figures 3 and 9A; Figure S2). These findings widely expand previous data indicating reduced NHP‐inducible *FMO1* expression in the *sard/cbp* double mutant (Nair et al., 2021). Notably, all of the hitherto identified NHP and SA‐pathway genes belong to the subcategory F. This emphasizes again that the SARD1/CBP60g transcriptional node enables the NHP‐triggered establishment of SAR both at the level of biosynthesis of the SAR signals NHP and SA and the level of NHP downstream action, thus corroborating the existence of an NHP‐driven and SARD1/CBP60g‐regulated feedback amplification mechanism for SAR activation (Figure 9B; Zeier, 2021). The characteristic that the Subgroup II consisting of SARD1/CBP60g‐dependent genes is significantly enriched in SA‐inducible genes further indicates a close interconnection of the action of SARD1/CBP60g and SA‐mediated gene expression (Figure 2C). More specifically, our analysis of the NHP transcriptional response suggest that SARD1/CBP60g may promote the responsiveness of SA‐inducible genes to NHP. Complimentarily, previous co‐expression and clustering analyses identified an SA‐regulated cluster of genes with enriched target sites for SARD1/CBP60g binding (Truman & Glazebrook, 2012). These findings indicate an important role for SA in fueling the suggested NHP‐ and SARD1/CBP60g‐mediated feedback loop that culminates in SAR establishment (Figure 2C; Bernsdorff et al., 2016; Nawrath & Métraux, 1999; Wildermuth et al., 2001).

On the other hand, yet functionally characterized genes encoding key enzymes of the Trp‐derived metabolism are exclusively found in the subcategory P, whose NHP‐triggered expression is not promoted or even negatively affected by SARD1/CBP60g (Figures 3 and 9A; Figure S2). Trp catabolism toward biosynthesis of indolic secondary metabolites in Arabidopsis begins with the conversion of Trp into the branch point intermediate indole‐3‐acetaldoxime (IAOx) by the two homologous cytochrome P450 monooxygenases CYP79B2 and CYP79B3 (Zhao et al., 2002). *CYP79B2* and *CYP79B3* transcripts exhibit high constitutive levels in leaves but are not elevated upon SAR induction, neither in the systemic leaf tissue of locally pathogen‐induced plants nor in the leaves of NHP‐treated plants (Figure 9A, Data S1; Bernsdorff et al., 2016; Yildiz et al., 2021). The Trp catabolic pathway is channeled into camalexin production by CYP71A13, which catalyzes a dehydration reaction from IAOx to IAN (Nafisi et al., 2007; Figure 9A). IAN is subsequently conjugated to glutathione, and the glutathione‐S‐transferase GSTF6 has been suggested to play a significant role in this conjugation step (Su et al., 2011). Ultimately, the strongly pathogen‐inducible CYP450 enzyme PAD3 (alias CYP71B15) catalyzes the two final steps of camalexin biosynthesis, the conversion of Cys‐IAN into camalexin with dihydrocamalexic acid as a reaction intermediate (Böttcher et al., 2009; Schuhegger et al., 2006; Zhou et al., 1999). In addition, the CYP71A13 homolog CYP71A12 contributes to stress‐inducible camalexin accumulation and was furthermore implicated in the formation of ICC, ICA and ICN from IAOx (Müller et al., 2015; Pastorczyk et al., 2020). Moreover, mutant analyses suggest that the aldehyde oxidase ARABIDOPSIS ALDEHYDE OXIDASE1 (AAO1) functions in the redox conversion of ICC to ICA (Böttcher et al., 2014), while the flavin‐dependent oxidoreductase FOX1 was shown to mediate generation of the indolic cyanide ICN (Rajniak et al., 2015). Remarkably, all genes coding for the above‐discussed enzymes of the Trp catabolic pathway (*PAD3, CYP71A13, CYP71A12, AAO1*, and *FOX1*) are, together with genes involved in indolic glucosinolate metabolism, significantly induced by NHP in a SARD1/CBP60g‐independent manner and thus belong to the gene subcategory P (Figures 3 and 9A, Figure S2).

In parallel with this series of biosynthetic genes involved in Trp‐derived metabolism, genes involved in the cellular processing of indoles (*PDR12*) and in the transcriptional regulation of the indolic pathway (*WRKY33* and *MYB51*) also showed SARD1/CBP60g‐independent elevation of transcript levels upon NHP treatment (Figure 3). *PDR12* codes for a plasma membrane‐resident ATP binding cassette transporter that was implicated in the cellular export of camalexin, and potentially other indolics, into the apoplast for protection against fungal invasion (He et al., 2019). Moreover, the pathogen‐inducible TF WRKY33 positively regulates the expression of several metabolic genes of the indole pathway and the accumulation of camalexin (Barco et al., 2019; Mao et al., 2011), and MYB51 is involved in the regulation of the stimulus‐triggered biosynthesis of indolic glucosinolates, ICC, and ICA (Frerigmann et al., 2016; Stahl et al., 2016). These examples underscore that the SARD1/CBP60g node does not promote indolic pathway activation, albeit tightly controlling the SAR‐related NHP and SA metabolic pathways. This does not rule out that individual regulatory genes expressed independently of SARD1/CBP60g do exert impact on SAR‐related metabolism, as exemplified by the positive impact of WRKY33 on Pip and NHP biosynthesis (Wang et al., 2018). Nevertheless, the bipartite function of the SARD1/CBP60g hub in the control of major pathogen‐inducible metabolic pathways is remarkable and distinguishes it from the EDS1/PAD4 regulatory node, which acts more comprehensively and boosts NHP, SA, and indolic metabolism together (Figure 9B; Feys et al., 2001; Hartmann et al., 2018; Jirage et al., 1999; Zhou et al., 1998).

### Many SARD1/CPB60g‐independent genes are responsive to H_2_O_2_

Our analysis of the NHP‐induced transcriptional response revealed significant differences in the abundance of regulatory features within the SARD1/CPB60g‐dependent subcategory F and the SARD1/CPB60g‐independent subcategory P genes. Notably, compared with the F subcategory, the P subcategory exhibits both an enrichment in H_2_O_2_‐responsive and a depletion in SA‐responsive genes (Figure 2D). The same tendency was observed for the Subgroup III genes that are NHP‐inducible in the *sard/cpb* double mutant but not in Col‐0 (Figure 2C). This suggests a significant role for ROS in the NHP‐triggered activation of SARD1/CPB60g‐independent genes in general and indolic pathway genes in particular (Figure 9B). Paradigm examples of strongly NHP‐inducible and ROS‐regulated genes of the P subcategory encode the blue‐copper‐binding protein BCB (SAG14), WRKY40, the zinc finger TF ZAT6, and the ubiquitin ligase CARBON/NITROGEN INSENSITIVE 1 (CNI1) (Figure 3; Desikan et al., 2001; Willems et al., 2016). In addition, these tendencies may indicate that functional SARD1/CBP60g dampens the responsiveness of H_2_O_2−_inducible genes to NHP.

Consistent with a function of ROS in the activation of the indolic pathway in Arabidopsis, ROS‐generating treatments such as silver nitrate application have been frequently used to trigger the accumulation of various indolic secondary metabolites in leaf tissue (Glawischnig, 2007; Müller et al., 2019; Schuhegger et al., 2006). Remarkably, the NAC TF ANAC042, which was identified as a regulator of ROS‐induced stress responses (Wu et al., 2012), is among the NHP‐induced subcategory P genes (Figure 3). ANAC042 was shown to physically interact with the promoters of the *PAD3* and *CYP71A12* genes and positively affect pathogen‐ and silver nitrate‐induced camalexin accumulation (Monsalvo et al., 2025; Saga et al., 2012). Moreover, MYB51 has been identified as a ROS‐responsive and indole glucosinolate‐regulating TF (Figure 3; Willems et al., 2016). A function of ROS in the activation of indolic metabolism is further suggested by the metabolic phenotype of Arabidopisis plants overexpressing the H_2_O_2_‐regulated protein kinase OXIDATIVE SIGNAL‐INDUCIBLE1 (OXI1), since these plants constitutively accumulate camalexin and other indolic compounds. Interestingly, when compromising NHP synthesis by introgression of *fmo1* or *ald1* mutant backgrounds into the OXI1 overexpressor lines, camalexin accumulation is significantly reduced, underscoring the above‐suggested interplay of ROS‐related and NHP signaling in indolic pathway activation (Rawat et al., 2023).

Although the critical genes for SAR activation are found in the SARD1/CPB60g‐dependent subcategory F genes that exhibit, on average, low H_2_O_2_ inducibility (Figure 2D), previous studies indicate that ROS participate in SAR establishment and NHP signal transduction. On the one hand, H_2_O_2_ generated by NADPH oxidases was proposed as a mobile signal that sulfenylates the TF CCA1 HIKING EXPEDITION (CHE), which was identified a decisive event in SAR establishment upstream of the action of NHP (Cao et al., 2024). On the other hand, ROS might also function downstream of NHP in SAR, since exogenous NHP resulted in enhanced diaminobenzidine staining in leaves, indicating NHP‐induced H_2_O_2_ accumulation (Li et al., 2023). Moreover, the NADPH oxidase genes *RESPIRATORY BURST OXIDASE HOMOLOG D* (*RBOHD*) and *RBOHF* were partially required for the execution of NHP‐induced immunity and NHP‐triggered accumulation of extracellular nicotinamide adenine dinucleotide (phosphate) [eNAD(P)], whose systemic accumulation is necessary for SAR and mediated through the L‐aspartate oxidase FLAGELLIN‐INSENSITIVE 4 (FIN4; Li et al., 2023). According to our RNA‐seq‐data, the transcript levels of *RBOHD*, but not of *RBOHF*, *CHE*, and *FIN4*, tended to increase upon NHP treatment (Figure S6). However, due to the rather modest increases in its expression levels, *RBOHD* did not fall into the defined Col^+^ category of NHP‐inducible genes (Figure 2). Nevertheless, the NHP‐induced increase in RBOHD transcript levels occurred to similar levels in Col‐0, *sard1*, *cbp60g*, and *sard/cbp* plants, suggesting a SARD1/CPB60g‐independency of NHP‐induced ROS formation (Figure S6). By contrast, previously published RNA‐seq data suggest that the NHP‐induced upregulation of *RBOHD* expression requires TGA TFs (Figure S6C; Yildiz et al., 2023).

### The bipartite action of SARD1/CPB60g directly manifests itself in the NHP‐mediated amplification of inducible plant defenses

Our previous studies showed that the transcriptional response to NHP in SAR‐induced plants primes plants for a boosted induction of a diverse array of defense responses during a challenge infection, and that the induced biosynthesis of SA and camalexin belongs to the most prominent of these primed responses (Bernsdorff et al., 2016; Löwe et al., 2023; Yildiz et al., 2021). As emphasized before, the SARD1/CBP60g node is essential for the NHP‐induced priming of pathogen‐triggered SA biosynthesis (Figure 8A). In stark contrast, the NHP‐triggered priming of *P. syringae*‐induced camalexin generation proceeded independently of functional SARD1/CBP60g and even proved to be negatively regulated by these TFs (Figure 8B). Therefore, the bipartite action of the SARD1/CBP60g node also manifests itself at the level of priming for enhanced plant responses to pathogens. In other words, plants may mediate priming of different defense responses by distinct molecular mechanisms.

### The dual function of SARD1/CPB60g is associated with distinct distributions of gene regulatory features

The evaluation of our RNA‐seq data revealed similarities and differences in the abundance of regulatory DNA elements in the SARD1/CPB60g‐dependent subcategory F and the SARD1/CPB60g‐independent subcategory P genes by use of two different motif analysis tools—the PTRMD algorithm and the TAIR motif finder (Figure 2E; Table S3; Reiser et al., 2022; Tian et al., 2020). On the one hand, TF‐motif analyses using both the PTRMD and TAIR algorithms predicted a strong over‐representation of WRKY target motifs in both of the F and P group genes (Figure 2E), including W‐box and WT‐box elements (Table S3; Song et al., 2023). In addition, the present and previous work indicate that a large number of WRKY TFs are strongly upregulated in Arabidopsis leaves upon induction of SAR by bacterial inoculation, application of NHP or treatment with the synthetic SAR inducer S‐methyl‐1,2,3‐benzothiadiazole‐7‐carbothioate (BTH), so that the WRKY family constitutes the most prominently activated class of TF in SAR‐induced tissue (Figure 3; Data S1; Bernsdorff et al., 2016; Wang et al., 2006; Yildiz et al., 2021). This wide upregulation of WRKYs during the SAR response and the concomitant enriched presence of WRKY‐targeted regulatory elements in the promoter regions of SAR genes supports the previously suggested notion that sequential WRKY‐supported transcriptional waves might operate in the course of SAR induction (Bernsdorff et al., 2016; Moore et al., 2011). Consistently, it was previously shown that WRKY18 controls the expression of a fraction of the BTH‐induced and NPR1‐regulated genes in Arabidopsis leaves (Wang et al., 2006). In addition, a recent time‐dependent analysis of the early transcriptional response to NHP revealed a sequential induction of early‐expressed, for the most part SA‐dependent genes that is followed by a later expression wave which is partially mediated by WRKY70 and involves genes with largely SA‐independent expression mode (Foret et al., 2025).

On the other hand, significant differences existed between the overall abundance of regulatory elements in the promoter regions of the F‐ and P‐ subcategory genes. The most striking differences predicted by the PTRMD algorithm involved the specific enrichment of regulatory elements targeted by bZIP‐ and C2H2‐TFs within the SARD1/CPB60g‐dependent F genes (Figure 2E). The bZIP family is subdivided into different subgroups, and their subgroup D comprises the TGA factors, of which the class II members (TGA2, TGA5, and TGA6) possess a key role in SAR and related responses downstream of NHP, while the class I members (TGA1 and TGA4) participate in the regulation of SA and NHP biosynthesis (Dröge‐Laser et al., 2018; Sun et al., 2018; Yildiz et al., 2023; Zhang et al., 2003). Preferred bZIP target sequences often consist of palindromic sequences with an ACGT‐core with adjacent nucleotides, such as the C‐box (GACGTC), the G‐box (CACGTG), and the A‐box (TACGTA) (Dröge‐Laser et al., 2018; Jakoby et al., 2002). Moreover, the TGACGT motif is specifically targeted by TGA TFs (Franco‐Zorrilla et al., 2014). Notably, while the C‐box and the TGACGT sequence turned out as the most strongly enriched hexamer motifs of the subcategory F genes, they are not enriched across the P‐subcategory genes (Table S3), further suggesting key roles of TGA factors in the expression of SARD1/CPB60g‐dependent SAR genes. This is consistent with the observation that SARD1/CPB60g and TGA factors act together in the regulation of SAR‐related gene expression (Sun et al., 2015, 2018).

The subcategory P gene *ZAT6* encodes a member of the C2H2‐TFs, for which target DNA elements were found enriched in the F subcategory (Figure 2E). Consistently, it was shown that ZAT6 binds to the motif TACAAT in several SAR‐related genes such as *EDS1*, *PAD4*, as well as several *PR* genes (Shi et al., 2014). This again exemplifies that SARD1/CPB60g‐independent P genes might also positively impact the expression of critical SAR‐regulatory genes that are primarily assembled in the SARD1/CPB60g‐dependent subcategory F. In addition, previous studies have established that SARD1 and CBP60g interact with the oligonucleotide sequence GAAATTTTGG *in vitro* and that a fragment thereof, GAAATTT, is enriched in the promoter regions of genes positively regulated by SARD1/CPB60g in *P. syringae*‐inoculated leaves (Wang et al., 2011; Zhang et al., 2010). However, no hexameric sequence inside the GAAATTT(TGG) stretch was identified by our current motif analysis, neither in the F nor in the P subcategory, in the context with the SAR‐related transcriptional response to NHP (Table S3).

### A broader role for the SARD1/CBP60g node in stress‐related metabolism and the balance of plant immunity

Notably, we observed that the selective influence of SARD1/CBP60g on *P. syringae*‐inducible metabolism is not restricted to the SA‐, NHP‐, and indole‐associated pathways (Figure 9A). As part of the metabolic reprogramming following bacterial infection, Arabidopsis leaves significantly alter their contents in several free amino acids, and accumulation of the NHP precursor Lys, the BCAAs Leu, Val, and Ile, and the AAAs Phe, Tyr, and Trp belong to the most significant changes in this regard (Návarová et al., 2012; Zeier, 2013). Although the biochemistry and regulation of the underlying amino acid‐related metabolic pathways are not yet comprehensively understood, our present results indicate a pronounced positive influence of the SARD1/CBP60g node on the accumulation of both the three BCAAs and the three AAAs. By contrast, SARD1/CBP60g did not affect the accumulation of Lys and had a partially negative impact on the leaf levels of Ser and Gly (Figures 6 and 9A). The contents of Ser and Gly might be influenced by the activities of serine hydroxymethyltransferases (SHMs), which catalyze the reversible interconversion of Ser and Gly (Nogués et al., 2022). Our RNA‐seq analysis identified *SHMT5* as an NHP‐inducible and SARD1/CBP60g‐promoted subcategory F gene (Figure 3). However, considering the existence of six other SHM isoforms that are not responsive to NHP and show in part strong basal expression (Data S1), it is far from clear whether the differential expression of *SHMT5* in Col‐0 and *sard/cbp* plants causes differences in Ser and/or Gly contents.

The increased leaf levels of Tyr following *P. syringae* inoculation may serve as an additional source for the biosynthesis of tocopherols, Tyr‐derived antioxidants that protect unsaturated membrane lipids from oxidative damage in the course of pathogen infection (Stahl et al., 2019). The tocopherol variant γ‐tocopherol accumulates to particularly high levels in *P. syringae*‐*inoculated* leaves. The current analysis indicates that the induced biosynthesis of γ‐tocopherol is, alongside its precursor amino acid Tyr, significantly promoted by the SARD1/CBP60g node (Figures 4 and 6), and a previous study furthermore showed that both Tyr and γ‐tocopherol accumulation are also boosted by the EDS1/PAD4 regulatory hub (Stahl et al., 2019). In an early biosynthetic step, tocopherol generation requires an aminotransferase reaction that converts Tyr into 4‐hydroxyphenylpyruvate (Mène‐Saffrané, 2017). Interestingly, our RNA‐seq analysis indicates the NHP‐induced upregulation of an annotated tyrosine aminotransferase gene, *TAT3*, which belongs to the subcategory F genes (Figure 3). The SARD1/CBP60g‐dependent expression of *TAT3* could thus in part explain the observed regulation of γ‐tocopherol biosynthesis, given that TAT3 would actually be involved in tocopherol‐related Tyr deamination. In contrast to γ‐tocopherol accumulation, generation of the unsaturated phytosterol stigmasterol proceeds independently from SARD1/CBP60g (Figure 4). Stigmasterol is generated from β‐sitosterol by a side chain desaturation reaction catalyzed by CYP710A1 (Griebel & Zeier, 2010; Morikawa et al., 2006). Consistent with the observed SARD1/CBP60g‐independent accumulation of stigmasterol, *CYP710A1* is expressed to similar levels in the leaves of Col‐0 and *sard/cbp* upon NHP treatment (Data S1).

The biological SAR response proves to be a highly appropriate solution to strengthen plant immunity when it is needed, and at the same time, ensures a time‐resolved balance between the investment of plants in growth and defense. First, SAR is not constitutively activated but induced by a primary localized pathogen encounter of leaves. Second, the thereby induced SAR state does not activate the plants' whole defense arsenal but rather primes them for a more massive defense capability if a secondary infection takes place (Figure 9; Löwe et al., 2023; Yildiz et al., 2021). And third, the SAR state is timely restricted and reversible, because the major SAR‐inducing metabolites NHP and SA are converted into inactive forms (Zeier, 2021). The glycosyltransferase UGT76B1 serves as a central hub for the regulation of a balanced immune activation because it catalyzes the conversion of the two major SAR‐activating metabolites NHP and SA to the immune‐inactive glucoside NHPG and SAG, which suppresses or terminates the SAR state (Bauer et al., 2021; Cai et al., 2021; Xu et al., 2025). Moreover, SA levels are also balanced via 2,3‐ and 2,5‐hydroxylation reactions catalyzed by the 2‐oxoglutarate‐dependent dioxygenases S3H and S5H (Zeilmaker et al., 2015; Zhang et al., 2013, 2017). Since NHP activates the key genes responsible for the metabolic inactivation, *UGT76B1, S3H*, and *S5H* (Figure 3; Yildiz et al., 2021), it is not only critical for the activation of SAR but also for the finalization of the SAR state. Both our metabolite analyses and our transcriptional data indicate that the SARD1/CBP60g node promotes this NHP‐induced termination of SAR (Figures 3, 4 and 9, Figures S4 and S5).

Negative regulation of SAR is also mediated by suppressors of SAR gene transcription. The three interacting NAC factors ANAC036, ANAC061, and ANAC090 negatively regulate NHP biosynthesis (Cai et al., 2024), and all of them represent NHP‐inducible genes (Data S1; Yildiz et al., 2021). The present RNA‐seq analysis also indicates that SARD1/CBP60g promote this negative regulatory unit because ANAC036 emerged as a subcategory F gene (Figure 3). Correspondingly, ANAC090 was identified as a target gene of SARD1 (Cai et al., 2024). In addition, CAMTA factors CAMTA1/2/3, which are not transcriptionally activated by NHP (Data S1), negatively regulate the expression of SA and NHP biosynthesis genes by modulating the expression of SARD1 and CBP60g (Sun et al., 2020), and the above‐discussed NHP‐inducible TF WRKY70 was shown to repress SARD1 in the absence of biotic stress by directly binding to its promoter (Zhou et al., 2018). These findings illustrate that the interplay of NHP and SARD1/CBP60g node is also involved in the suppression of SAR activation under stress‐free conditions and, in an interplay with NHP, in the termination of SAR in previously induced plants. Moreover, SAR establishment, in parallel with the biosynthesis of the inducers SA and NHP, is vulnerable to high ambient temperatures, and CBP60g and SARD1 were suggested to be involved in the events that control these temperature effects on immune signaling (Shields et al., 2025). Also, SARD1/CBP60g has been identified as an important node in the crosstalk between drought and biotic stress signaling (Choudhary & Senthil‐Kumar, 2022). These findings indicate a broad role of SARD1/CBP60g in integrating different stress‐related responses in plants.

## MATERIALS AND METHODS

### Plant cultivation

*Arabidopsis thaliana* plants were cultivated in individual pots with a mixture of soil (Substrat BP3; Klasmann‐Deilmann), sand and vermiculite (8:1:1) under 60% relative humidity in Polyklima® growth cabinets (L3 TDL dual) equipped with true daylight LED panels. Seeds were stratified in liquid nitrogen before transferring to soil and kept under a lid until they germinated. Ten to twelve days after sowing, the seedlings were separated into individual pots and watered three times a week. The growth cabinets had a day/night cycle of 10 h/14 h with day conditions from 9 AM to 19 PM. The LEDs were set to 25% warm white and 9.4% cold white, and temperatures during the day and night periods were set to 21°C and 18°C, respectively. For experiments, approximately 5‐week‐old plants of the accession Col‐0 [Nottingham Arabidopsis Stock Centre (NASC) ID: N1092; wild‐type background line for all of the mutants] and the following homozygous mutant lines were used: *sard1*‐*2* (*sard1*; Salk_052422; Wang et al., 2011), *cbp60g*‐*1* (cbp60g; Salk_023199; Wang et al., 2011), *sard1*‐*2*/*cbp60g*‐*1* (*sard/cbp*; N72191), *npr1*‐*3* (N3802), and *sid2*‐*1* (Nawrath & Métraux, 1999).

### Cultivation of bacterial strains and inoculation

For plant inoculations with *P. syringae* pv. *maculicola* ES4326 (*Psm*; Zeier et al., 2004) or bioluminescent *Psm* expressing the luxCDABE operon from *Photorhabdus luminescens* (*Psm lux*; Fan et al., 2008), overnight cultures of bacteria were prepared in King's B as previously described (Bernsdorff et al., 2016; Gruner et al., 2018). After about 16 h of constant shaking, the culture was washed three times with 10 mM MgCl_2_ and diluted to optical densities at 600 nm (OD_600_) of 0.005 for *Psm* and 0.001 for *Psm lux*, respectively. The bacterial suspensions (or a mock‐treatment solution of 10 mM MgCl_2_) were infiltrated between 9 and 11 AM into the abaxial side of the leaves with a needleless syringe. *Psm* was used for all experiments in which metabolite contents and gene expression were analyzed. *Psm lux* was used in resistance assays to determine bacterial numbers in leaves.

### Resistance assays

To assess the biological induction of SAR, three lower rosette leaves of a given Arabidopsis plant were inoculated with *Psm* (OD 0.005) to induce SAR or mock‐treated (1° treatment). Forty‐eight hours later, three upper leaves of pre‐treated plants were challenge‐inoculated with *Psm lux* (OD 0.001). Another 2.5 d later, leaf disks were punched out of the upper, 2°‐challenged leaves, and bacterial numbers determined as relative light units (rlu) per cm^2^ with a Sirius luminometer (Berthold Detection Systems GmbH, Pforzheim/Germany) as detailed in Gruner et al. (2018). Generally, 6 to 12 plants per genotype received inducing or mock‐ pre‐treatments, so that in general, 18 to 36 replicate leaf disk samples were analyzed (Figure 1).

For the assessment of SAR establishment by exogenous NHP (synthesized as described in Hartmann et al., 2018), we infiltrated three lower rosette leaves of a given plant with an aqueous solution of 1 mM NHP or with water as a control treatment, challenge‐inoculated three upper leaves 24 h later with *Psm lux* (OD 0.001), and determined bacterial numbers in these leaves 2.5 days post‐inoculation as described above (Yildiz et al., 2023).

To assess the local induction of resistance by exogenous SA (Sigma‐Aldrich, S5922), a 0.5 mM SA solution in water containing 0.1% EtOH was infiltrated into three leaves per plant. Control plants were treated likewise with a 0.1% EtOH solution. Four hours later, the pre‐treated leaves were challenge‐inoculated with *Psm lux* (OD 0.001) and bacterial numbers assayed 2.5 days later as outlined above (Yildiz et al., 2023).

### Plant treatments for assessment of metabolite contents and gene transcript levels

To analyze *P. syringae*‐induced local changes in metabolite or transcript levels, three leaves of a plant were infiltrated with a suspension of *Psm* (OD 0.005) or with the mock‐control solution (10 mM MgCl_2_). The three treated leaves were harvested at the indicated times after treatment (usually 12, 24, or 48 hpt) and combined for one replicate sample. For the analysis of systemic responses, three lower leaves of a plant were treated as described above, and three distant, upper leaves were harvested at 48 hpt and combined for one replicate sample.

To determine UV light‐induced metabolic changes in Arabidopsis leaves, plants were uniformly exposed for 30 min to UV‐B light by use of a shelf rack equipped with a UV lamp (model VL‐115.BL, Vilber‐Lourmat, France) containing T‐15 M light tubes with a maximum emission at 312 nm and 15 W power. The UV‐irradiation was generally performed between 10 and 12 AM, and the distance of plant rosettes to the light source was 40 cm. After irradiation, plants were returned to the conventional growth conditions for 24 h, and three leaves per plant were then harvested and combined to obtain one replicate sample.

To assess NHP‐induced priming of SA and camalexin biosynthesis, a previously established protocol was used (Figure 8A,B; Yildiz et al., 2021). Ten milliliters of a 1 mM aqueous NHP solution was pipetted onto the soil of individually cultivated Arabidopsis plants to induce priming. As a control treatment, 10 ml of water was used instead. One day after the first treatment, three rosette leaves were inoculated with *Psm* (OD 0.005) for bacterial challenge or mock‐infiltrated with 10 mM MgCl_2_. The treated leaves of a given plant were harvested after 12 h, and combined for one replicate sample to assess metabolite contents. The leaves of a third set of plants were not treated at all after the soil treatment, and three leaves of comparable age harvested as described.

In addition, we also assessed the direct effects of NHP on SA accumulation by infiltrating three leaves of a given plant with 1 mM NHP solution or with water, and combined the treated leaves 48 h later for one replicate sample to determine metabolite contents. In total, six biological replicates were analyzed (Figure 8C).

### Metabolite analysis

The levels of most of the reported metabolites were determined by gas chromatography–mass spectrometry (GC–MS)‐based qualitative and quantitative analysis of trimethylsilylated analytes as described previously (Yildiz et al., 2023). The harvested leaf samples were immediately shock‐frozen in liquid N_2_ and ground to a fine powder using a pre‐chilled ball mill. Approximately 50 mg of the frozen, powdered leaf material was extracted twice with 1 ml of extraction buffer [MeOH/10 mM NaPO_4_ (80:20, v/v, pH 6.0)] by shaking the samples at 4° C and 150 rpm for 10 min. In the first extraction step, the extraction buffer contained a defined set of internal standards (1 μg each). Six hundred microliters aliquots of the combined extracts were evaporated to dryness and derivatized with 20 μL N‐methyl‐N‐trimethyl‐silyltrifluoroacetamide (MSTFA) containing 1% trimethylchlorosilane (v/v) in 20 μL pyridine and 60 μL of hexane for 30 min at 70°C. The samples were diluted 1:4 in hexane and then analyzed with an Agilent 7890A/5975C GC–MS system equipped with a Phenomenex ZB‐35 (30 m × 0.25 mm × 0.25 μm) capillary column and MSD ChemStation software version E.02.01.1177 (Agilent Technologies) as detailed in Yildiz et al. (2023). For quantitative analysis, selected ion chromatograms of analytes and related internal standards were integrated [analyte (*m/z*) related to internal standard (*m/z*)]: Pip (*m/z* 156) related to deuterium‐labeled D_9_‐Pip (*m/z* 165); NHP (*m/z* 172) related to D_9_‐NHP (*m/z* 181); SA (*m/z* 267) related to D_4_‐SA (*m/z* 271); NHPG, NHPGE (*m/z* 172), SAG (*m/z* 267), and SGE (*m/z* 193) all related to salicin (*m/z* 268); camalexin (*m/z* 272), ICA (*m/z* 246), and Trp (*m/z* 202) all related to indole‐3‐propionic acid (*m/z* 202); Val (*m/z* 144), Leu (*m/z* 158), Ile (*m/z* 158), Phe (*m/z* 218), Tyr (*m/z* 218), Gly (*m/z* 174), and Ser (*m/z* 218) all related to norvaline (*m/z* 144); Lys (*m/z* 317) related to D_8_‐Lys (*m/z* 325); γ‐Toc (*m/z* 488) related to tocol (*m/z* 460). Furthermore, the ratios of the peak areas of stigmasterol (*m/z* 484) and β‐sitosterol (*m/z* 486) were determined.

The levels of the indolic compounds ICC and I3N were assessed by LC/q‐TOF‐MS analysis (Scholten et al., 2024). For sample work‐up, about 50 mg of frozen, powdered leaf material was extracted with 600 μL of extraction buffer [MeOH:H_2_O (80:20, v/v)], to which 100 ng of dehydrojasmonic acid was added as internal standard, by thoroughly vortexing and further extracting for 10 min on a rotatory shaker at 4°C. After centrifugation at 14000 rpm, the supernatant was removed and the pellet extracted again with pure extraction buffer. The solvent of the combined extracts was evaporated at 30°C using a ScanSpeed vacuum centrifuge (Labogene ApS). The dry residue was redissolved in 100 μl of LC–MS‐grade MeOH:H_2_O (80:20, v/v), the solution filtered through a nylon centrifugal filter (VWR, 516–0233), and 5 μl of the filtrate injected into a 1260 Infinity II Prime LC equipped with an InfinityLab Poroshell 120 EC‐C18 column (3.0 × 100 mm i.d., 2.7 μm particle size). The LC system was coupled to a 6540‐quadrupole time‐of‐flight mass spectrometer with a dual electrospray ionization (ESI) source (Agilent Technologies). The parameters of LC separation and MS analysis are detailed in Scholten et al. (2024). The MS analysis was performed in the negative ionization mode. For quantification, the peak areas of specific extracted ion chromatograms ([M‐H]^−^ ions ±20 ppm: ICC, 144.0455; I3N, 141.0458) were integrated and related to both the peak area of the internal standard (DHJA, ([M‐H]^−^ 211.1340)) and the sample FW.

### Analysis of gene transcript levels by RT‐qPCR

To analyze gene transcript levels by RT‐qPCR, we isolated total RNA from about 50 mg of frozen leaf samples using my‐Budget RNA‐Magic reagent (Bio‐Budget Technologies GmbH) following the manufacturer's instructions. One microgram isolated RNA was treated with DNAse I, RNase free (Thermoscientific) at 37°C for 30 min and inactivated by 25 mM EDTA during an incubation at 65°C for 10 min. The mRNA was reverse transcribed into cDNA by using GoScript™ reverse transcription mix and oligo(dT) primer (Promega Corporation) as described by the manufacturer. The 2.5 μl of the cDNA was amplified in 10 μl reaction volume using the GoTaq® qPCR Master Mix (Promega Corporation). *POLYPYRIMIDINE TRACT*‐*BINDING PROTEIN 1* (*PTB1*) was chosen as a reference gene since its expression is non‐responsive to *P. syringae* inoculation (Czechowski et al., 2005). The qPCR was performed in duplicates in a Rotor‐Gene Q apparatus (Qiagen) using the fast‐cycling program from the GoTaq® qPCR Master Mix protocol. The sequences of the used gene‐specific primers are listed in Table S2. Data evaluation was performed according to the ΔΔC_T_ method as previously described (Návarová et al., 2012). The expression values of a particular gene were related to the mean of the wild‐type control values (Figure 7).

### Statistical analysis of data obtained from metabolite, RT‐qPCR‐based gene expression and bacterial growth assays

The number of biological replicates, applied statistical tests, and related information are detailed in each of the figure legends. Outliers were detected with the Dixon Test in R‐Studio version 4.4.1 and statistical analysis performed with the SPSS® statistical software version 27 (IBM® Corporation). In case of the bacterial growth assays of *Psm lux* the log_10_‐transformed measuring values were compared in an analysis of variance (ANOVA) and a post hoc Tukey's HSD test (significance level *P* < 0.05; Hartmann et al., 2018). A non‐parametric one‐way ANOVA according to Kruskal–Wallis with stepwise step‐down comparisons (significance level *P* < 0.05) was performed on the non‐transformed metabolite and RT‐qPCR data to test their statistical differences in data subsets.

### Genome‐wide analyses of the NHP transcriptional response by RNA sequencing

The whole‐genome transcriptional response of rosette leaves of Col‐0, *sard1*, *cbp60g*, and *sard/cbp* plants toward NHP was analyzed by RNA sequencing by a previously established protocol, which is described in detail in Yildiz et al. (2023). Plants of the different genotypes were either supplied with 10 ml of 1 mM NHP or with 10 ml of water as control treatment via the soil. Twenty‐four hour later, 18 rosette leaves from six different plants (three leaves per plant) per genotype and treatment were shock‐frozen in liquid nitrogen and pooled for one biological replicate sample. In total, three independent replicate experiments were performed. Thus, three biologically replicate samples per treatment and plant genotype were obtained (Table S1).

RNA was isolated from about 50 mg of powdered, frozen leaf material as described above. An additional clean‐up step using the Plant RNeasy Extraction Kit (Qiagen) was applied. RNA was treated on‐column (Qiagen) and in solution with RNA‐free DNAse (New England Biolabs) before quantification for transcriptome analysis. The procedures for the quantification of total RNA, RNA quality insurance, Illumina® RNA sequencing, and data analysis were performed according to the protocol detailed in Yildiz et al. (2023). Data analyses on obtained FastQ‐format files were conducted with CLC Genomics Workbench (version 22.0.2; Qiagen, Venlo, NL). The data were mapped against the *Arabidopsis thaliana* TAIR10.42 genome sequence. After grouping of samples according to their respective genotype and treatment, the statistical differential expression was determined using the Differential Expression for RNA‐Seq tool (version 2.7). The resulting *P*‐values were corrected for multiple testing by FDR and Bonferroni‐correction.

The RNA‐seq analysis covered 27 655 Arabidopsis genes in total (Data S1). To evaluate the data, genes lacking expression (cpm = 0) in any of the 24 RNA samples were excluded, resulting in a total of 23 365 analyzed, leaf‐expressed genes (Figure 2). Genes with FDR‐adjusted *P* values ≤0.005 between cpm values of NHP‐ versus water‐treated samples of one genotype and fold changes (FC) ≥3.0 (≤0.33) for the means of cpm values were defined as Col^+^, *sard* ^+^, *cbp* ^+^, and *sard/cbp* ^+^ (Col^−^, *sard* ^*−*^, *cbp* ^*−*^, and *sard/cbp* ^*−*^) genes, respectively. The 558 NHP‐induced Col‐0 genes (Col^+^) were further divided into Subgroup I [significantly upregulated in *sard/cbp* (FDR ≤0.005, FC ≥3.0)] and Subgroups II (not significantly upregulated in *sard/cbp*). Moreover, Subgroup III consisted of genes with significant upregulation in *sard/cbp* but not in Col‐0 (Figure 2A,C). Another classification procedure considered Col‐0‐to‐*sard/cbp*‐ratios of mean cpm values of NHP‐treatment samples to subdivide the Col^+^ gene group into two subcategories (F* with ratios >1.5, P* with ratios ≤1.5; Figure 2D). Furthermore, F* genes with significant differences (*P* < 0.02) between the NHP‐Col‐0 and the NHP‐*sard/cbp*‐samples were defined as strongly SARD1/CPB60g‐promoted subcategory F genes, while P* genes with Col‐0‐to‐*sard/cbp*‐ratios of mean cpm values ≤1.2 were defined as P‐subcategory genes that essentially lack SARD1/CPB60g‐promoted expression (Figure 2D).

To estimate the percentages of SA‐, JA‐, and H_2_O_2_‐inducible genes within the different gene groups, gene lists derived from previous microarray studies were merged into the present gene tables using Microsoft Excel® (Davletova et al., 2005; Goda et al., 2008; Thibaud‐Nissen et al., 2006; Data S1). Fisher's exact test corrected according to Benjamini and Hochberg (1995) was used to determine whether gene categories were significantly enriched or depleted in specific gene characteristics. All of the defined gene groups depicted in Figure 2 are given, together with the corresponding gene expression data and statistical values, in the individual spreadsheets of Data S1.

Transcription factor‐motif differential enrichment analysis was performed using the Plant Transcriptional Regulatory Map database (https://plantregmap.gao‐lab.org/tf_enrichment.php; Tian et al., 2020). Moreover, the abundance of hexamer motifs in the 1000 bp upstream regions of the subcategory F and P genes was examined using the TAIR motif finder tool (https://v2.arabidopsis.org/tools/bulk/motiffinder/index.jsp). Significantly enriched motifs compared to abundances across the whole‐genome gene set were identified with a Fisher exact test (*P* < 0.005).

## AUTHOR CONTRIBUTIONS

MP and MG conducted stress‐related plant experiments, resistance assays, metabolite analysis, and gene expression analysis. PP and KK provided the platform for and performed Illumina® RNA sequencing. JZ and MP evaluated the metabolite and RNA‐seq data in detail and elaborated the manuscript figures and tables. JZ secured funding, conceived the research, and wrote the manuscript.

## CONFLICT OF INTEREST

The authors declare that they have no conflict of interest.

## Supporting information


**Figure S1.** Requirement and redundant action of the transcription factors SARD1 and CBP60g in the establishment of Arabidopsis acquired resistance in leaves induced by soil treatment with N‐hydroxypipecolic acid (NHP). Individual Col‐0, *sard1*‐*2*, *cbp60g*‐*1*, or *sard1*‐*2*/*cbp60g*‐*1* (*sard/cbp*) plants were supplied with 10 ml of an aqueous solution of 1 mM NHP or with 10 ml of H_2_O via the cultivation soil as a 1° treatment. One day later, plants were syringe‐infiltrated in three rosette leaves with a suspension of *Psm lux* (OD 0.001), and bacterial bioluminescence assessed at 2.5 days post‐inoculation. Bars show the mean ± SD of log_10_‐transformed relative light units (rlu) per cm^2^ leaf area of 15–18 leaf replicates. Gray circles represent individual data points of replicates. Different letters indicate significant differences (*P* < 0.05, ANOVA and post hoc Tukey HSD test). Related to Figure 1A.
**Figure S2.** Expression values of selected NHP‐inducible genes of the F and P subcategories in Col‐0, *sard1*, *cpb60g*, and *sard/cbp* plants, as determined by RNA sequencing. Mean cpm expression values (± SD) of NHP‐treatment‐ and H_2_O‐control samples are given in bar graph format. Individual cpm values resulting from the three independent experiments are super‐imposed (gray circles). (A) The 116 subcategory F genes show significantly attenuated NHP‐induced expression in *sard/cbp* and involve NHP‐ and SA‐pathway genes. They belong to subclass I (significantly induced by NHP in *sard/cbp*) or II (not significantly induced by NHP in *sard/cbp*), as indicated in brackets. (B) The 119 subcategory P genes show wild‐type‐like or enhanced NHP‐induced expression in *sard/cbp* and include genes involved in the biosynthesis of Trp‐derived defense metabolites. They all belong to subclass I. (C) Thirty‐six genes are significantly NHP‐induced in *sard/cbp* plants but not in Col‐0 (subclass III). The expression pattern of the *DTX18* gene is depicted as an example. Related to Figures 2 and 3, and Table S1.
**Figure S3.** Levels of SAR‐related defense metabolites in the *Psm*‐ or mock‐ (10 mM MgCl_2_) inoculated leaves of Col‐0 wild type, *sard1*, *cpb60g*, and *sard/cbp* plants at 24 h post‐treatment (hpt). Bars represent the means ± SD of three to five biological replicates. Individual data points are super‐imposed on the bar graphs (gray circles). Different letters denote significant differences (*P* < 0.05, Kruskal–Wallis H‐test). (A) Salicylic acid (SA), SA‐β‐glucoside (SAG), SA glucose ester (SGE). (B) N‐hydroxypipecolic acid (NHP), NHP‐*N*‐*OH*‐glucoside (NHPG), NHP glucose ester (NHPGE). Related to Figure 5A.
**Figure S4.** Levels of SAR‐related defense metabolites in distant (upper) leaves of Col‐0, *sard1*, *cpb60g*, and *sard/cbp* plants infiltrated in their lower leaves with a suspension of *Psm* or with mock‐solution. Bars represent the means ± SD of three to four biological replicates. Individual data points are super‐imposed on the bar graphs (gray circles). Different letters denote significant differences (*P* < 0.05, Kruskal–Wallis H‐test). (A) Salicylic acid (SA), SA‐β‐glucoside (SAG), SA glucose ester (SGE). (B) N‐hydroxypipecolic acid (NHP), NHP‐*N*‐*OH*‐glucoside (NHPG), NHP glucose ester (NHPGE). Related to Figure 5B.
**Figure S5.** Levels of SAR‐related defense metabolites in the leaves of Col‐0, *sard1*, *cpb60g*, and *sard/cbp* after exposing the plants to a UV‐B light stress period of 30 min. Control plants were left untreated. Bars represent the means ± SD of four to five biological replicates. Individual data points are super‐imposed on the bar graphs (gray circles). Different letters denote significant differences (*P* < 0.05, Kruskal–Wallis H‐test). (A) Salicylic acid (SA), SA‐β‐glucoside (SAG), SA glucose ester (SGE). (B) N‐hydroxypipecolic acid (NHP), NHP‐*N*‐*OH*‐glucoside (NHPG), NHP glucose ester (NHPGE). Related to Figure 5C.
**Figure S6.** Expression values of selected ROS‐related genes, as determined by RNA sequencing.
(A, B) RNA‐seq‐data of the current study (Data S1). Mean cpm expression values (± SD) of NHP‐treatment‐ and H_2_O‐control samples are given in bar graph format. Individual cpm values resulting from the three independent experiments are super‐imposed (gray circles). (A) *RESPIRATORY BURST OXIDASE HOMOLOG D* (*RBOHD*). (B) *RBOHF*, *FLAGELLIN*‐*INSENSITIVE 4* (*FIN4*), and *CCA1 HIKING EXPEDITION* (*CHE*). Please note that none of the genes belong to the here‐defined NHP‐upregulated genes in any of the genotypes (Figure 2A), since FDR‐adjusted *P*‐values from comparison of NHP‐ and H_2_O‐samples were >0.05, and NHP/H_2_O‐ratios of mean cpm values were <3.0. However, for *RBOHD* (but not for the other three genes), a tendential upregulation by NHP was discernible, as suggested by the values of the non‐corrected *P*‐values of NHP‐ and H_2_O‐sample comparisons, which are given above the bars (see also Data S1). (C) Leaf *RBOHD* expression levels of Col‐0 and *tga2/tga5/tga6* (*tga2/5/6*) triple mutant plants, as assessed by the RNA‐seq study of Yildiz et al. (2023). Mean cpm expression values (± SD) of NHP‐treatment‐ and H_2_O‐control samples, as well as non‐corrected *P*‐values are given as indicated above.
**Table S1.** Experimental setup to compare the transcriptional response of Col‐0, *sard1*, *cpb60g*, and *sard/cbp* plants to NHP by RNA sequencing. The transcriptional response of Arabidopsis Col‐0 (wild type), *sard1*‐*2*, *cbp60g*‐*1*, and *sard1*‐*2*/*cbp60g*‐*1* plants to NHP was investigated in three independent experiments by RNA sequencing. Five‐week‐old plants were supplied via the soil with 10 ml of 1 mM NHP solution (equal to a dose of 10 μmol) or with 10 ml of H_2_O. For each experiment, three full‐grown leaves from six different plants were harvested 24 h after the respective treatment and pooled to generate one biological replicate. The implementation of three independent experiments thus yielded three biologically independent samples per treatment and plant genotype. RNA‐seq analysis of the resulting 24 samples was performed as described in the main text. The respective sample ID of each replicate is indicated in the table and matches the file annotations of the raw data files (FastQ format) accessible under https://doi.org/10.60534/zxve3‐smd73. The results are provided in Excel‐Format (Data S1) and illustrated in Figures 2 and 3 and Figure S2.
**Table S2.** Primers used for qPCR‐analyses.
**Table S3.** Motif analysis in the promoter regions of genes of the F and P subcategories. The occurrence of six base sequence motifs in the 1000‐bp upstream regions of the 116 genes of subcategory F and the 119 genes of subcategories P were compared with the abundance of the hexamer motifs across all (34 187) genes in the genomic set using the TAIR motif finder tool (https://v2.arabidopsis.org/tools/bulk/motiffinder/index.jsp). A Fisher exact test with *P* < 0.005 was used to define significantly enriched motifs in the gene subcategories. The identified hexamer sequences, their reverse complemented sequences, their occurrence (absolute numbers) in the whole‐genomic set, their occurrence (absolute numbers) in the F‐ and P‐subcategory genes, their fold enrichment (relative occurrence in subcategory / relative occurrence in genomic set) in the F and P subcategories, and the *P*‐values of the Fisher exact test are given. (A) Hexamer sequences selectively enriched in the promoter regions of subcategory F genes. (B) Hexamer sequences enriched in the promoter regions of both subcategory F and P genes. (C) Hexamer sequences selectively enriched in the promoter regions of subcategory P genes.

**Data S1.** Full RNA‐seq dataset of the transcriptional response to NHP of Col‐0, *sard1*‐*2*, *cbp60g*‐*1* and *sard1*‐*2*/*cbp60g*‐*1* plants. Individual spread sheets of the Excel file contain the different gene groups depicted in Figure 2, as well as the full set of RNA‐covered Arabidopsis genes (27655).

## Data Availability

To provide general accessibility to the obtained RNA‐seq data and ensure data FAIRness, we utilized the DataPLANT personal assistance network and its tool stack, centered around the DataHUB (Weil et al., 2023). The original FastQ‐format files of the RNA‐seq study are thus available under DOI: https://doi.org/10.60534/zxve3‐smd73.
