# Supplementary material for: SARD1 and CBP60g redundantly regulate N‐hydroxypipecolic acid‐ and salicylic acid‐dependent systemic immunity but are dispensable for the activation of indolic defense metabolism
Source: Plant J. 2026 Sep 21;127(6):e71130. doi: 10.1111/tpj.71130 (PMC13594504; doi:10.1111/tpj.71130)
Supplement: Supplementary file 1 [file TPJ-127-0-s002.pdf]

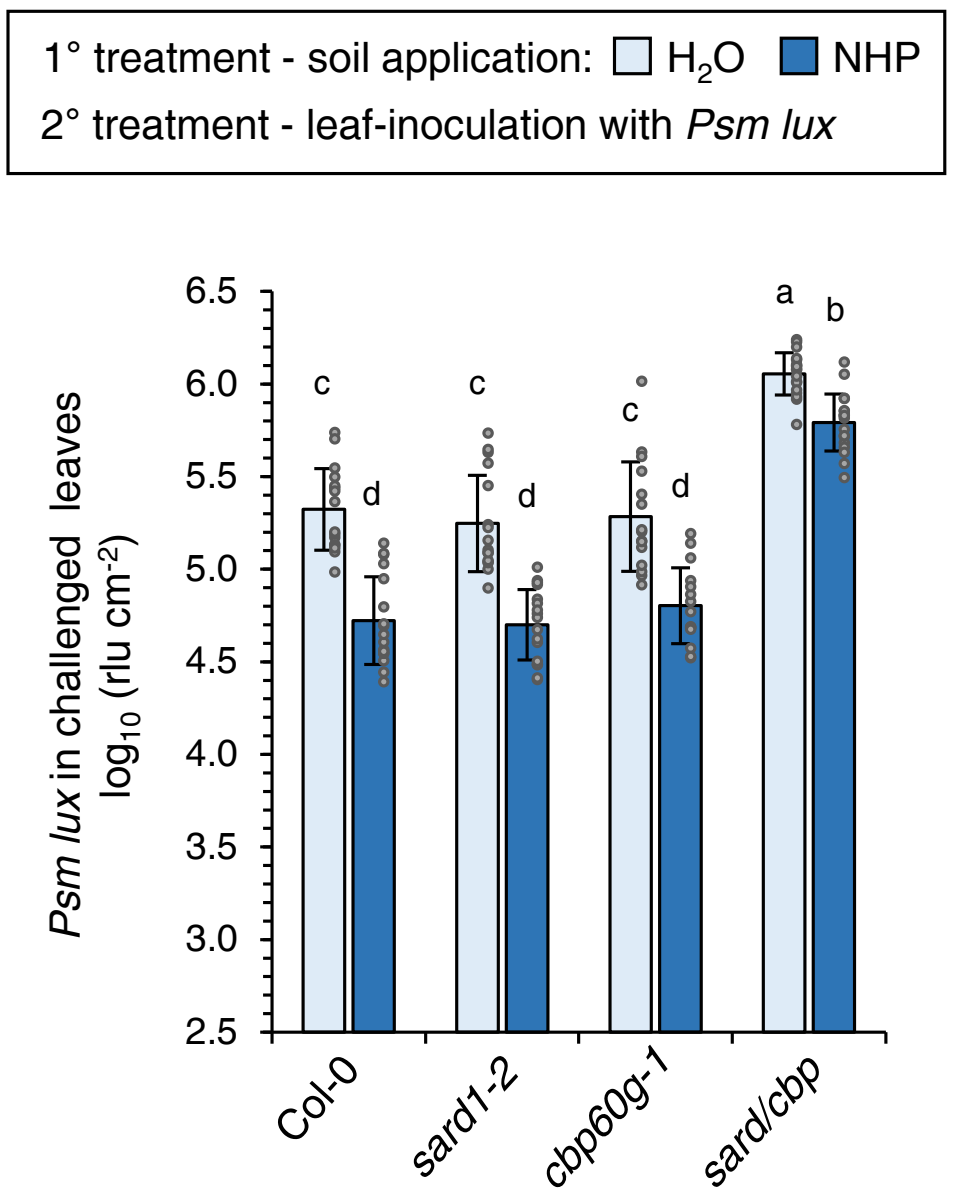

Figure S1. Requirement and redundant action of the transcription factors SARD1 and CBP60g in the establishment of Arabidopsis acquired resistance in leaves induced by soil treatment with N-hydroxypipecolic acid (NHP).

Individual Col-0, *sard1-2*, *cbp60g-1* or *sard1-2*/*cbp60g-1* (*sard/cbp*) plants were supplied with 10 ml of an aqueous solution of 1 mM NHP or with 10 ml of H_2O via the cultivation soil as a 1° treatment. One day later, plants were syringe-infiltrated in three rosette leaves with a suspension of *Psm lux* (OD 0.001), and bacterial bioluminescence assessed at 2.5 days post inoculation. Bars show the mean ± SD of $\log_{10}$-transformed relative light units (rlu) per cm^2 leaf area of 15-18 leaf replicates. Grey circles represent individual data points of replicates. Different letters indicate significant differences ($p < 0.05$, ANOVA and post hoc Tukey HSD test).

Related to Figure 1A.

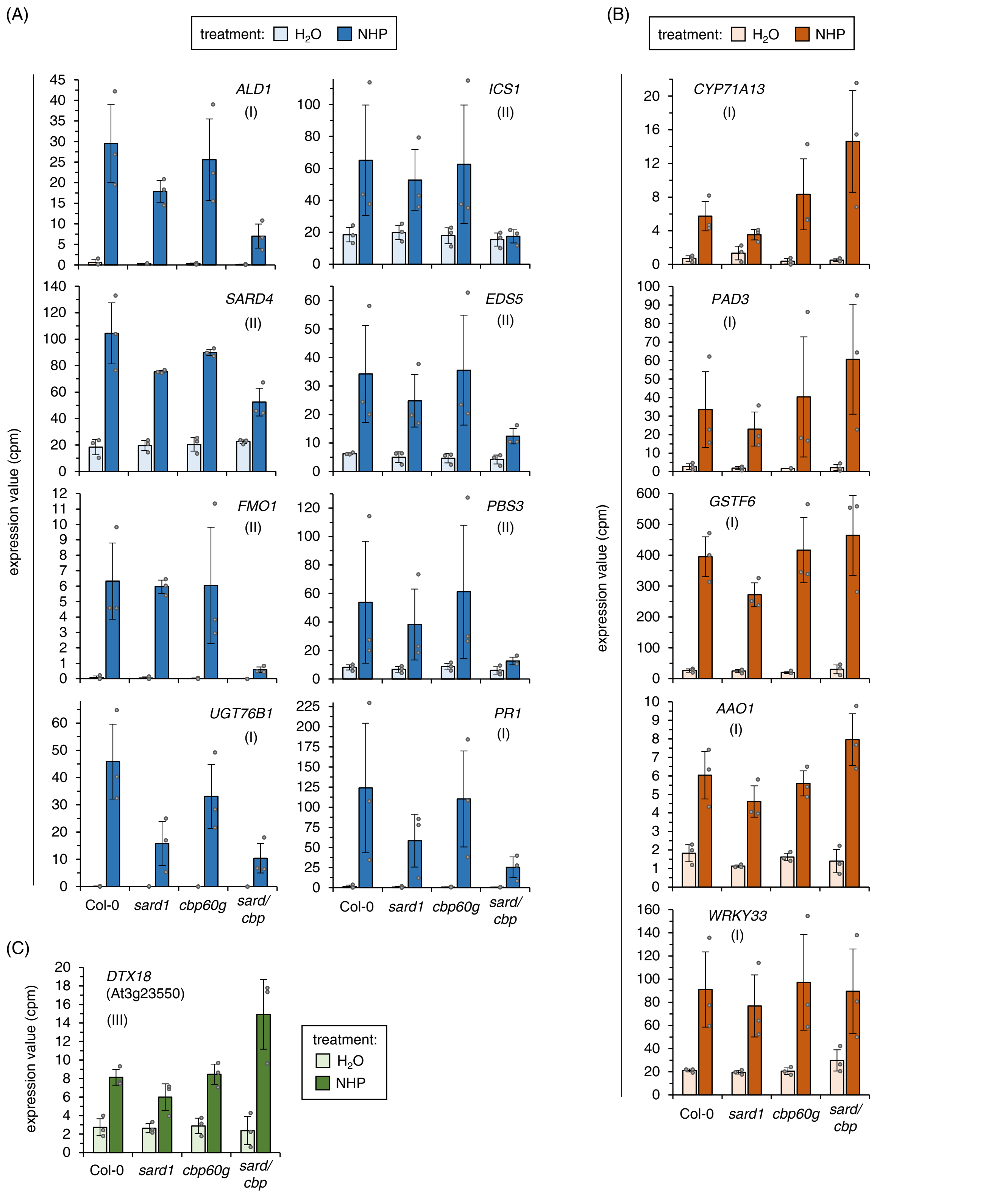

Figure S2. Expression values of selected NHP-inducible genes of the F and P subcategories in Col-0, *sard1*, *cpb60g* and *sard/cbp* plants, as determined by RNA sequencing.

Mean cpm expression values (± SD) of NHP-treatment- and H_2O-control samples are given in bar graph format. Individual cpm values resulting from the three independent experiments are super-imposed (grey circles).

(A) The 116 subcategory F genes show significantly attenuated NHP-induced expression in *sard/cbp* and involve NHP- and SA-pathway genes. They belong to subclass I (significantly induced by NHP in *sard/cbp*) or II (not significantly induced by NHP in *sard/cbp*), as indicated in brackets.

(B) The 119 subcategory P genes show wildtype like or enhanced NHP-induced expression in *sard/cbp* and include genes involved in the biosynthesis of Trp-derived defense metabolites. They all belong to subclass I.

(C) 36 genes are significantly NHP-induced in *sard/cbp* plants but not in Col-0 (subclass III). The expression pattern of the *DTX18* gene is depicted as an example.

Related to Figures 2, 3, and Table S1.

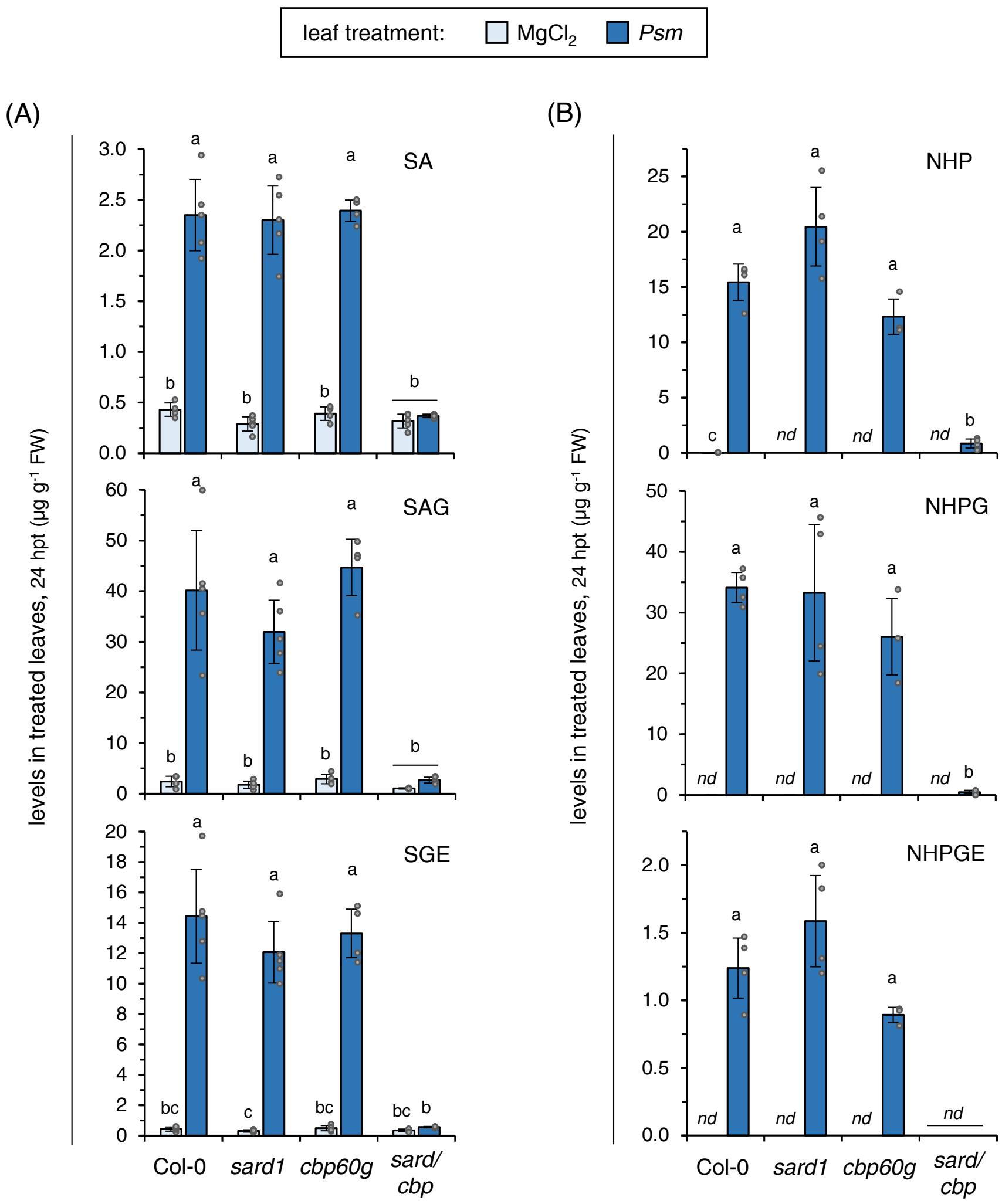

Figure S3. Levels of SAR-related defense metabolites in the *Psm*- or mock- (10 mM $MgCl_2$) inoculated leaves of Col-0 wildtype, *sard1*, *cpb60g* and *sard/cbp* plants at 24 h post treatment (hpt). Bars represent the means ± SD of three to five biological replicates. Individual data points are super-imposed on the bar graphs (grey circles). Different letters denote significant differences ($p < 0.05$, Kruskal-Wallis H test).

A) Salicylic acid (SA), SA-β-glucoside (SAG), SA glucose ester (SGE).

B) N-hydroxypipecolic acid (NHP), NHP-*N-OH*-glucoside (NHPG), NHP glucose ester (NHPGE).

Related to Figure 5A.

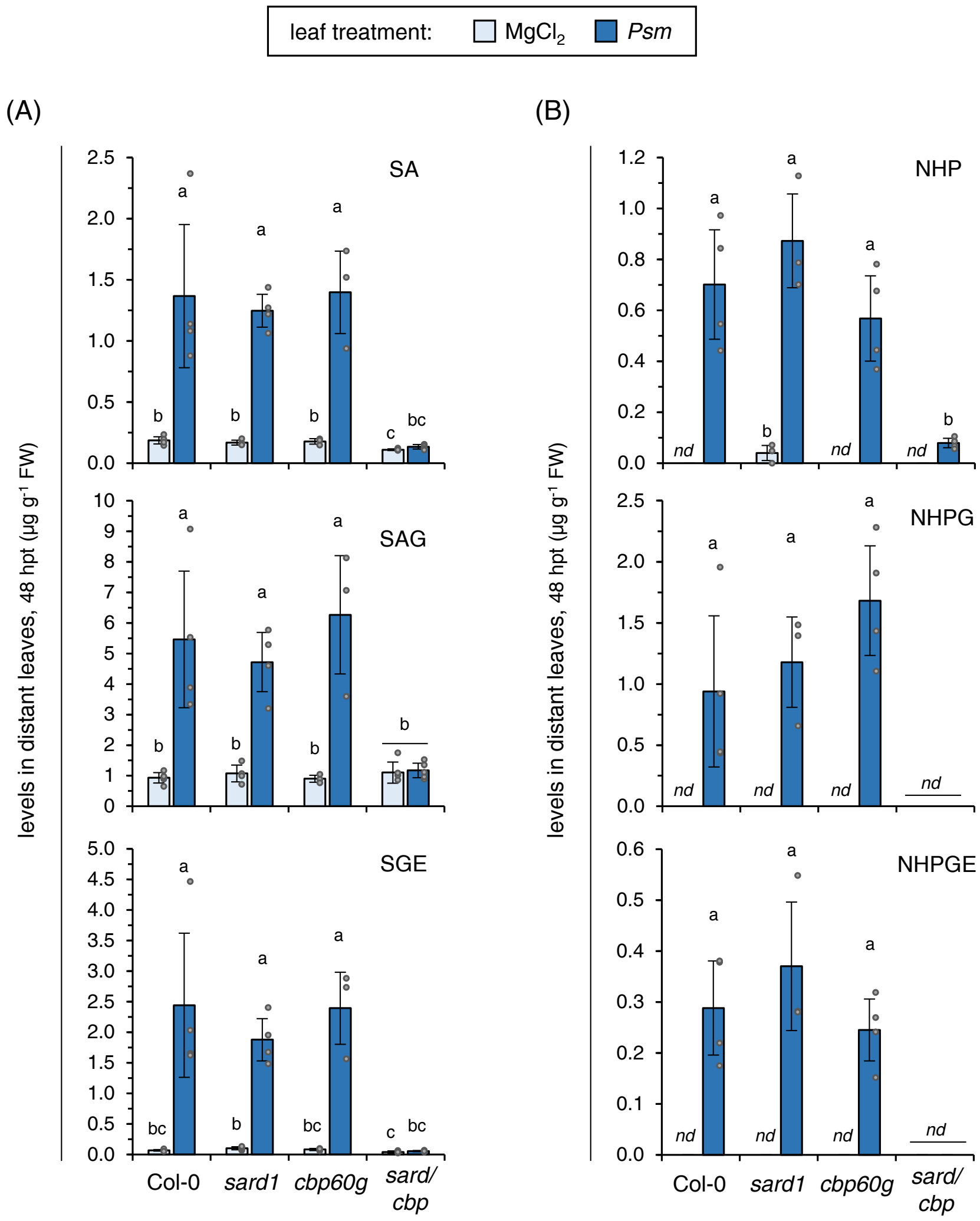

Figure S4. Levels of SAR-related defense metabolites in distant (upper) leaves of Col-0, *sard1*, *cpb60g* and *sard/cbp* plants infiltrated in their lower leaves with a suspension of *Psm* or with mock-solution. Bars represent the means ± SD of three to four biological replicates. Individual data points are super-imposed on the bar graphs (grey circles). Different letters denote significant differences ($p < 0.05$, Kruskal-Wallis H test).

A) Salicylic acid (SA), SA-β-glucoside (SAG), SA glucose ester (SGE).

B) N-hydroxypipecolic acid (NHP), NHP-*N-OH*-glucoside (NHPG), NHP glucose ester (NHPGE).

Related to Figure 5B.

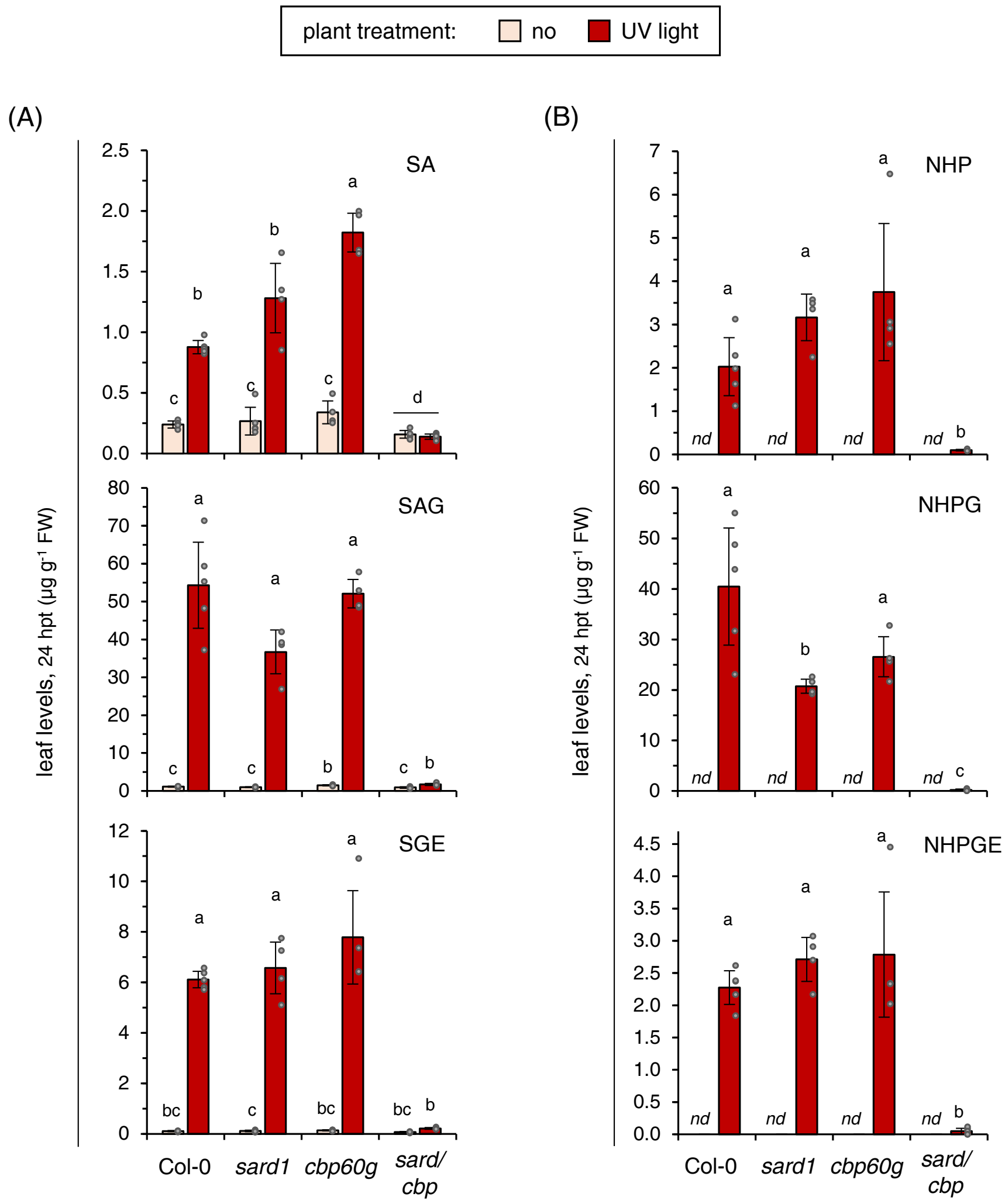

Figure S5. Levels of SAR-related defense metabolites in the leaves of Col-0, *sard1*, *cpb60g* and *sard/cbp* after exposing the plants to a UV-B light stress period of 30 min. Control plants were left untreated. Bars represent the means ± SD of four to five biological replicates. Individual data points are super-imposed on the bar graphs (grey circles). Different letters denote significant differences ($p < 0.05$, Kruskal-Wallis H test).

A) Salicylic acid (SA), SA-β-glucoside (SAG), SA glucose ester (SGE).

B) N-hydroxypipecolic acid (NHP), NHP-*N-OH*-glucoside (NHPG), NHP glucose ester (NHPGE).

Related to Figure 5C.

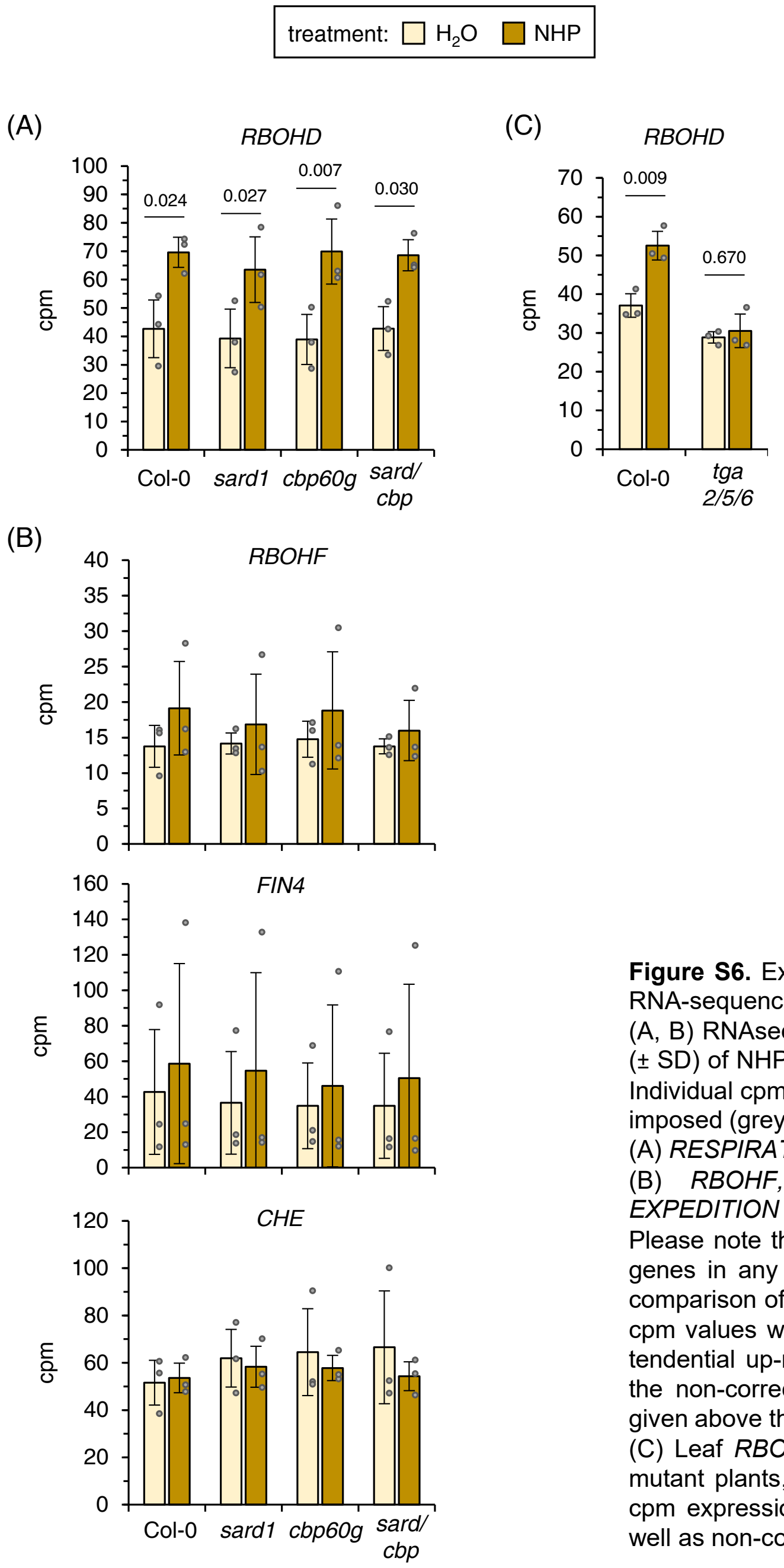

Figure S6. Expression values of selected ROS-related genes, as determined by RNA-sequencing.
(A, B) RNAseq-data of the current study (Data S1). Mean cpm expression values (± SD) of NHP-treatment- and H_2O-control samples are given in bar graph format. Individual cpm values resulting from the three independent experiments are superimposed (grey circles).
(A) *RESPIRATORY BURST OXIDASE HOMOLOG D (RBOHD).*
(B) *RBOHF, FLAGELLIN-INSENSITIVE 4 (FIN4),* and *CCA1 HIKING EXPEDITION (CHE).*
Please note that none of the genes belong to the here-defined NHP-upregulated genes in any of the genotypes (Figure 2A), since FDR-adjusted *P* values from comparison of NHP- and H_2O-samples were > 0.05, and NHP/H_2O-ratios of mean cpm values were < 3.0. However, for *RBOHD* (but not for the other 3 genes), a tendential up-regulation by NHP was discernible, as suggested by the values of the non-corrected *P* values of NHP- and H_2O-sample comparisons, which are given above the bars (see also Data S1).
(C) Leaf *RBOHD* expression levels of Col-0 and *tga2/tga5/tga6* (*tga2/5/6)* triple mutant plants, as assessed by the RNA-seq study of Yildiz et al. (2023). Mean cpm expression values (± SD) of NHP-treatment- and H_2O-control samples, as well as non-corrected *P* values are given as indicated above.

File name (FastQ)	Sample ID	Genotype	Treatment	Experiment	Replicate	Harvest	N° of leaves (Leaves x plant)
Col-0 H_2O – 1	1	Col-0	H_2O	1	R1	24h	18 (3 x 6)
Col-0 NHP – 1	2	Col-0	NHP	1	R1	24h	18 (3 x 6)
sard1-2 H_2O – 1	3	*sard1-2*	H_2O	1	R1	24h	18 (3 x 6)
sard1-2 NHP – 1	4	*sard1-2*	NHP	1	R1	24h	18 (3 x 6)
cbp60g-1 H_2O – 1	5	*cbp60g-1*	H_2O	1	R1	24h	18 (3 x 6)
cbp60g-1 NHP – 1	6	*cbp60g-1*	NHP	1	R1	24h	18 (3 x 6)
sard1-2/cbp60g-1 H_2O – 1	7	*sard1-2/cbp60g-1*	H_2O	1	R1	24h	18 (3 x 6)
sard1-2/cbp60g-1 NHP – 1	8	*sard1-2/cbp60g-1*	NHP	1	R1	24h	18 (3 x 6)
Col-0 H_2O – 2	9	Col-0	H_2O	2	R2	24h	18 (3 x 6)
Col-0 NHP – 2	10	Col-0	NHP	2	R2	24h	18 (3 x 6)
sard1-2 H_2O – 2	11	*sard1-2*	H_2O	2	R2	24h	18 (3 x 6)
sard1-2 NHP – 2	12	*sard1-2*	NHP	2	R2	24h	18 (3 x 6)
cbp60g-1 H_2O – 2	13	*cbp60g-1*	H_2O	2	R2	24h	18 (3 x 6)
cbp60g-1 NHP – 2	14	*cbp60g-1*	NHP	2	R2	24h	18 (3 x 6)
sard1-2/cbp60g-1 H_2O – 2	15	*sard1-2/cbp60g-1*	H_2O	2	R2	24h	18 (3 x 6)
sard1-2/cbp60g-1 NHP – 2	16	*sard1-2/cbp60g-1*	NHP	2	R2	24h	18 (3 x 6)
Col-0 H_2O – 3	17	Col-0	H_2O	3	R3	24h	18 (3 x 6)
Col-0 NHP – 3	18	Col-0	NHP	3	R3	24h	18 (3 x 6)
sard1-2 H_2O – 3	19	*sard1-2*	H_2O	3	R3	24h	18 (3 x 6)
sard1-2 NHP – 3	20	*sard1-2*	NHP	3	R3	24h	18 (3 x 6)
cbp60g-1 H_2O – 3	21	*cbp60g-1*	H_2O	3	R3	24h	18 (3 x 6)
cbp60g-1 NHP – 3	22	*cbp60g-1*	NHP	3	R3	24h	18 (3 x 6)
sard1-2/cbp60g-1 H_2O – 3	23	*sard1-2/cbp60g-1*	H_2O	3	R3	24h	18 (3 x 6)
sard1-2/cbp60g-1 NHP - 3	24	*sard1-2/cbp60g-1*	NHP	3	R3	24h	18 (3 x 6)

Table S1. Experimental setup to compare the transcriptional response of Col-0, *sard1*, *cpb60g* and *sard/cbp* plants to NHP by RNA-sequencing.

The transcriptional response of Arabidopsis Col-0 (wildtype), *sard1-2*, *cbp60g-1* and *sard1-2/cbp60g-1* plants to NHP was investigated in three independent experiments by RNA-sequencing. 5-week-old plants were supplied via the soil with 10 ml of 1 mM NHP solution (equal to a dose of 10 µmol) or with 10 ml of H_2O. For each experiment, 3 full-grown leaves from 6 different plants were harvested 24 h after the respective treatment and pooled to generate one biological replicate. The implementation of three independent experiments thus yielded three biologically independent samples per treatment and plant genotype. RNA-seq analysis of the resulting 24 samples was performed as described in the main text. The respective sample ID of each replicate is indicated in the table and matches the file annotations of the raw data files (FastQ format) accessible under https://doi.org/10.60534/zxve3-smd73. The results are provided in Excel-Format (Data S1) and illustrated in Figures 2, 3, and S2.

Gene			Primer sequence (5' to 3')
At2g13810	*ALD1*	Forward primer	GTGCAAGATCCTACCTTCCCGGC
		Reverse primer	CGGTCCTTGGGGTCATAGCCAGA
At2g30770	*CYP71A13*	Forward primer	ACATAGTTGAATCTTTCCCAAAGCACAATC
		Reverse primer	AAATTACCGGGATCTCGAACGGTGG
At1g19250	*FMO1*	Forward primer	TCTTCTGCGTGCCGTAGTTTC
		Reverse primer	CGCCATTTGACAAGAAGCATAG
At1g74710	*ICS1*	Forward primer	TTCTGGGCTCAAACACTAAAAC
		Reverse primer	GGCGTCTTGAAATCTCCATC
At3g26830	*PAD3*	Forward primer	GGCTGAAGCGGTCATAAGAG
		Reverse primer	TCCAGGCTTAAGATGCTCGT
At5g13320	*PBS3*	Forward primer	TGCCTGCTCGAGTCGCAACC
		Reverse primer	TGGACTAAGCCACAGAGCAAATGGC
At2g14610	*PR1*	Forward primer	GTGCTCTTGTTCTTCCCTCG
		Reverse primer	GCCTGGTTGTGAACCCTTAG
At1g43190	*PTB*	Forward primer	GATCTGAATGTTAAGGCTTTTAGCG
		Reverse primer	GGCTTAGATCAGGAAGTGTATAGTCTCTG
At3g11340	*UGT76B1*	Forward primer	CTTTACAAGAGACTAAGGCAG
		Reverse primer	CACACCTATCTGTAACTTATCCC

Table S2. Primers used for qPCR-analyses.

	6mer /	reverse compl.	number in genome set	number in subcat. F	fold enrichment in subcat. F	Fisher Exact test (*P* value)	number in subcat. P	fold enrichment in subcat. P	Fisher Exact test (*P* value)	remarks
**A**	GACGTC	GACGTC	5170	52	2.96	0.00006	28	1.56	0.0690	C-Box (bZIP); ACGT core
	TGACGT	ACGTCA	9025	70	2.29	0.00005	38	1.21	0.5751	(bZIP/TGA); ACGT core; contains TGAC
	GTGACG	CGTCAC	5945	43	2.13	0.00025	27	1.30	0.3093	contains TGAC
	TAGCGT	ACGCTA	4299	29	1.99	0.00037	13	0.87	1.0000	
	GTCACT	AGTGAC	10948	64	1.72	0.00030	40	1.05	0.4057	contains TGAC
	GACTTG	CAAGTC	14893	87	1.72	0.00310	66	1.27	0.1206	
	GATACG	CGTATC	6513	38	1.72	0.00397	21	0.93	1.0000	
	AGTCTT	AAGACT	22001	124	1.66	0.00050	106	1.38	0.0055	
	CACGTT	AACGTG	9452	51	1.59	0.00397	34	1.03	0.4489	ACGT core
	GGTTCC	GGAACC	7671	41	1.58	0.00133	21	0.79	0.7276	
	GTCAAG	CTTGAC	12807	68	1.56	0.00223	58	1.30	0.4257	contains TGAC
	TTAACG	CGTTAA	12500	66	1.56	0.00097	38	0.87	0.5443	
	GTCTTT	AAAGAC	28468	141	1.46	0.00036	128	1.29	0.0966	
	GTACTT	AAGTAC	14269	68	1.40	0.00379	54	1.09	0.3272	
	GTATTT	AAATAC	38095	163	1.26	0.00477	135	1.02	0.3889	
	TTAAGT	ACTTAA	30258	121	1.18	0.00266	114	1.08	0.4602	
	TTTACA	TGTAAA	44387	159	1.06	0.00434	151	0.98	0.4227	
	TTTTTA	TAAAAA	123751	434	1.03	0.00427	444	1.03	0.1009	
**B**	TTGACT	AGTCAA	25580	138	1.59	0.00276	136	1.53	0.0001	W-box; contains TGAC
	TGACTT	AAGTCA	24145	127	1.55	0.00017	122	1.45	0.0023	contains TGAC
	GACTTT	AAAGTC	24570	128	1.54	0.00076	133	1.56	0.0003	WT-box
	TAATAT	ATATTA	68424	251	1.08	0.00153	302	1.27	0.0004	
**C**	CGACGT	ACGTCG	5289	25	1.39	0.32257	35	1.90	0.0019	ACGT core
	TGACCG	CGGTCA	4974	18	1.07	0.58301	31	1.79	0.0017	contains TGAC
	GTCAAT	ATTGAC	19630	74	1.11	0.34786	105	1.54	0.0039	contains TGAC
	CGTATT	AATACG	10854	44	1.19	0.13870	55	1.46	0.0005	
	GTTGAC	GTCAAC	14367	72	1.48	0.01306	72	1.44	0.0016	contains TGAC
	TAATTA	TAATTA	79126	272	1.01	0.18390	354	1.29	0.0035	
	TTTAAT	ATTAAA	86294	301	1.03	0.03937	372	1.24	0.0006	
	GTAATT	AATTAC	33535	117	1.03	0.57170	144	1.23	0.0020	
	ATTAGT	ACTAAT	36250	157	1.28	0.05547	154	1.22	0.0018	
	TTTTGA	TCAAAA	75950	260	1.01	0.88834	318	1.20	0.0000	
	TTCAAC	GTTGAA	28702	91	0.93	0.51270	120	1.20	0.0040	
	GGTTTC	GAAACC	20034	52	0.76	0.07434	82	1.18	0.0021	
	AAGTTT	AAACTT	58730	224	1.12	1.00000	240	1.17	0.0004	
	TAATTG	CAATTA	35671	112	0.93	0.70161	139	1.12	0.0044	

Table S3. Motif analysis in the promoter regions of genes of the F and P subcategories.
The occurrence of six base sequence motifs in the 1000 bp upstream regions of the 116 genes of subcategory F and the 119 genes of subcategories P were compared to the abundance of the hexamer motifs across all (34187) genes in the genomic set using the TAIR motif finder tool (https://v2.arabidopsis.org/tools/bulk/motiffinder/index.jsp). A Fisher exact test with $P < 0.005$ was used to define significantly enriched motifs in the gene subcategories. The identified hexamer sequences, their reverse complemented sequences, their occurrence (absolute numbers) in the whole genomic set, their occurrence (absolute numbers) in the F and P subcategory genes, their fold-enrichment (relative occurrence in subcategory / relative occurrence in genomic set) in the F and P subcategories, and the *P* values of the Fisher exact test are given.

A) Hexamer sequences selectively enriched in the promoter regions of subcategory F genes.
B) Hexamer sequences enriched in the promoter regions of both subcategory F and P genes.
C) Hexamer sequences selectively enriched in the promoter regions of subcategory P genes
